# The Convergence Coefficient across Political Systems

**DOI:** 10.1155/2013/653035

**Published:** 2013-12-10

**Authors:** Maria Gallego, Norman Schofield

**Affiliations:** ^1^Center in Political Economy, Washington University, 1 Brookings Drive, Saint Louis, MO 63130, USA; ^2^Department of Economics, Wilfrid Laurier University, 75 University Avenue West, Waterloo, ON, Canada N2L 3C5

## Abstract

Formal work on the electoral model often suggests that parties or candidates should locate themselves at *the electoral mean*. Recent research has found no evidence of such convergence. In order to explain nonconvergence, the stochastic electoral model is extended by including estimates of electoral valence. We introduce the notion of a convergence coefficient, *c*. It has been shown that high values of *c* imply that there is a significant centrifugal tendency acting on parties. We used electoral surveys to construct a stochastic valence model of the the elections in various countries. We find that the convergence coefficient varies across elections in a country, across countries with similar regimes, and across political regimes. In some countries, the centripetal tendency leads parties to converge to the electoral mean. In others the centrifugal tendency dominates and some parties locate far from the electoral mean. In particular, for countries with proportional electoral systems, namely, Israel, Turkey, and Poland, the centrifugal tendency is very high. In the majoritarian polities of the United States and Great Britain, the centrifugal tendency is very low. In anocracies, the autocrat imposes limitations on how far from the origin the opposition parties can move.

## 1. Introduction

Work on modeling elections has often assumed that the policy space was restricted to one dimension or that there were at most two parties [1, 2]. The extensive formal literature on electoral competition has typically been based on the assumption that parties or candidates adopt positions in order to win and has inferred that parties will converge to the electoral median, under deterministic voting in one dimension, or to the electoral mean in stochastic models.

In this paper we offer a formal stochastic model of elections that emphasizes the importance of the idea of *valence* and use this notion to provide an explanation of why vote maximizing political leaders in some countries will not adopt convergent policy positions at the mean of the electoral distribution. In the standard spatial model, candidate *positions* matter to voters. However, as Stokes [3, 4] has emphasized, the nonpolicy evaluations, or *valences*, of candidates by the electorate are just as important. (See also Clarke et al.   [5, 6], Scotto et al. [7], and Clarke et al. [8].)

The main objective of this paper is to examine whether parties locate close to or far from the electoral mean (the electoral mean is the mean of voters ideal policies dimension by dimension) of various countries. We use Schofield's [9] stochastic electoral model as a unifying framework that allows us to compare parties positions across different political systems. In this model, parties respond to their partisan constituencies after taking into account the anticipated electoral outcome and the positions of other parties. Voters decisions depend on parties' locations and on the party's valence, the voters' *overall common* evaluation of the ability of a party leader to provide good governance. Using this model we examine whether parties converge to electoral mean in several elections in various countries under different political systems and use convergence, or the lack thereof, to classify political systems.

To examine whether parties converge to the electoral mean in each country in a particular election, we test whether any party has an incentive to stay or move away from the electoral mean to increase its vote share. In formal voting theory, it is usual to define a “Nash equilibrium” as a vector of party positions with the property that no party may make a unilateral move so as to increase its vote share. We use a variant of this concept, that is a “local Nash equilibrium” (LNE) where we consider only *marginal* moves from the position. One of the standard results in formal theory is the *mean voter theorem*, where the “Nash equilibrium” of a spatial voting game under vote maximization is one where all parties position themselves at the electoral mean. (For variants of the theorem see Enelow and Hinich [10–12]). We call such a vector the *electoral mean*.

To study each party's best response to the electoral situation they face, we use the results presented in Schofield [9]. Schofield identifies a *convergence coefficient*, denoted *c*, whose value determines whether vote maximizing parties converge or not to the electoral mean. This coefficient depends on various parameters of the model. In particular it depends on the *competence valences* of the party leaders. Using *j* ∈ *P* = 1,…, *p* to denote the parties, the valence of party *j*, *λ*_*j*_, essentially measures the electoral perception of the “quality” of *j*, the voters' *overall common* evaluation of the ability of *j*'s leader to provide good governance. The valence terms, ***λ*** = (*λ*_1_,…, *λ*_*p*_), are assumed to be independent of the party's positions and can be estimated as the intercept term in the appropriate stochastic model of the voter utility function. As Sanders et al. [13] comment, valence theory is based on the assumption that “voters maximize their utilities by choosing the party that is best able to deliver policy success.” These valence terms measure the *bias* in favor of one another of the party leaders [14].

The convergence coefficient, *c*, also depends on the weight that voters give to the policy differences they have with the various parties *β*. Lastly, *c* depends on the variance/covariance matrix of the electoral distribution, *σ* ^2^. By its construction, *c* ≡ *c*(***λ***, *β*, *σ* ^2^) is dimensionless and thus independent of the units of measurement of the various parameters. We use the convergence coefficient to compare results across elections and countries and to classify political systems.

The convergence coefficient is a summary measure that provides an estimate of the centrifugal or centripetal forces acting on the parties. The *Valence Theorem*, presented in Section 2 (see Schofield [9] for the proof of this result) shows that if the policy space is two-dimensional and if *c*(***λ***, *β*, *σ* ^2^) < 1, then the sufficient condition for convergence to the mean has been met and the “local Nash Equilibrium” (LNE) (the set of such local Nash Equilibria contains the set of Nash Equilibria) is one where all parties locate at the electoral mean. On the other hand, if *w* is the dimension of the policy space and *c*(***λ***, *β*, *σ* ^2^) ≥ *w*, then the LNE, if it exists, will be one where at least one party will have an incentive to diverge from the electoral mean in order to maximize its vote share. Thus, the necessary condition for convergence to the mean is that *c*(***λ***, *β*, *σ* ^2^) < *w*.

In essence, a high empirical convergence coefficient of an election is a convenient measure of the electoral incentive of a small, or low valence, party to move away from the electoral mean to its core constituency position. We can interpret a high value of the convergence coefficient as a measure of the *centrifugal* tendency exerted on parties pulling them away from the electoral mean. The convergence coefficient is therefore a convenient, simple, and intuitive way to examine whether parties will have an incentive to locate close to, or far from, the electoral mean. We will show that there is a strong connection between the values of the convergence coefficient and the nature of the political system under which parties operate.

We used preelection polls to study elections in several countries operating under different political regimes. The factor analysis done on preelection surveys showed that for all elections the policy space was two-dimensional, except in Azerbaijan were it was one-dimensional. The position of voters along this two-dimensional space were then estimated and their voting intentions used to estimate party positions. We then ran a multinomial logit (MNL) model for the election using the estimated party and voter positions. The intercept of the MNL model gives the valences of each party/leader. Following Schofield's [9] formal model, we rank parties according to their valence. Using these MNL estimates we calculate the convergence coefficient of the election and examine whether the party with the lowest valence has an incentive to locate close to or far from the electoral origin.

When comparing the convergence coefficients across countries we observe that in countries with proportional representation the convergence coefficient is high and that in countries with plurality systems or in anocracies it is low. Thus, suggesting that we can use the valence theorem and its associated convergence result to classify electoral systems.

The convergence coefficients for the 2005 and 2010 elections in the UK were not significantly different from 1, meeting the necessary condition for convergence to the mean. For the 2000, 2004, and 2008 US presidential elections, the convergence coefficient is significantly below 1 in 2000 and 2004 thus meeting the sufficient and thus necessary condition for convergence and not significantly different from 1 in 2008, only meeting the necessary condition for convergence. We suggest that the centrifugal tendency in the majoritarian polities like the United States and the United Kingdom is very low.

In contrast, the convergence coefficient gives an indication that the centrifugal tendency in Israel, Poland, and Turkey is very high. In these proportional representation systems with highly fragmented polities the convergence coefficients are significantly greater than 2 failing to meet the necessary condition for convergence to the electoral mean.

In the anocracies of Georgia, Russia, and Azerbaijan, where the President/autocrat dominates and controls who can run in legislative elections, the convergence coefficient is not significantly different from the dimension of the policy space (2 for Georgia and Russia and 1 for Azerbaijan), failing the necessary condition for convergence. While the analysis Georgia and Azerbaijan show that not all parties converge to the mean, in Russia it is likely that they did. Thus, in Russia opposition parties found it difficult to diverge from the mean. Note that convergence in anocracies may not generate a stable equilibrium as any change in the valence of the autocrat and the opposition may cause parties to diverge from the mean and may even lead to popular uprising that bring about changes in the governing parties such as in Georgia in previous elections or in the Arab revolutions.

We can also classify polities using the *effective vote number* and the *effective seat number.* (Fragmentation can be identified with the *effective number*. That is, let *H*_*v*_ (the Herfindahl index) be the sum of the squares of the relative vote shares and let *env* = *H*_*v*_^−1^ be the *effective number of party vote strength*. In the same way we can define *ens* as the effective number of party seat strength using shares of seats. See Laakso and Taagepera [15].) We examine how these two measures of fragmentation relate to the convergence coefficient for the polities we consider. The effective vote or seat numbers give an indication of the difficulty inherent in interparty negotiation over government. These two measures do not, however, address the fundamental aspect of democracy, namely, the electoral preferences for policy. Since convergence involves both preferences, in terms of the electoral covariance matrix and the effect of the electoral system, we argue that the Valence Theorem and the associated convergence coefficient allow for a more comprehensive way of classifying polities and political systems precisely because it is derived from the fundamental characteristics of the electorate. That is, while we can use the effective vote and seat number to identify which polities are fragmented, the Valence Theorem and the convergence result can help us understand why parties locate close to or far from the electoral mean and how, under some circumstances, these considerations lead to political fragmentation.

The next section presents Schofield's [9] stochastic formal model of elections and implications it has for convergence to the mean.  Section 3.1 applies the model to the elections to two plurality polities: The United States and the United Kingdom. In Section 3.2 we apply the model to polities using proportional electoral systems, namely, Israel, Turkey, and Poland.  Section 3.3 considers the convergence coefficients for three “anocracies:” Azerbaijan, Georgia, and Russia. Comparisons between different fragmentation measures and the convergence coefficient are examined in Section 4.  Section 5 concludes the paper. In the appendix we estimate the confidence intervals for the convergence coefficient as well as determining whether the low valence party has an incentive to deviate from the electoral mean.

## 2. The Spatial Voting Model with Valence

Recent research on modelling elections has followed earlier work by Stokes [3, 4] and emphasized the notion of valence of political candidates. As Sanders et al. [13] comment, valence theory extends the spatial or Downsian model of elections by considering not just the policy positions of parties but also the parties' rival attractions in terms of their perceived ability to handle the most serious problems that face the country. Thus, voters maximize their utilities by choosing the party that they think is best able to deliver policy success.

Schofield and Sened [16] have also argued that Valence relates to voters' judgments about positively or negatively evaluated conditions which they associate with particular parties or candidates. These judgements could refer to party leaders' competence, integrity, moral stance or “charisma” over issues such as the ability to deal with the economy and politics.

Valence theory has led to a considerable theoretical literature on voting based on the assumption that valence plays an important role in the relationship between party positioning and the votes that parties receive. (Ansolabare and Snyder [17], Groseclose [18], Aragones and Palfrey [19, 20], Schofield [21], Schofield et al. [22], Miller and Schofield [23], Schofield and Miller [24], Peress [25]) Empirical work, based on multinomial logit (MNL) methods, has also shown the importance of electoral judgements in analyses of elections in the United States and the United Kingdom. (Clarke et al. [8, 26–28], Schofield [29], Schofield et al. [30, 31], Scotto et al. [7]) These empirical models of elections have a “probabilistic” component. That is, they all assume that “voter utility” is partly “Downsian” in the sense that it is based on the distance between party positions and voter preferred positions and partly due to valence. The estimates of a party's valence is assumed to be subject to a “stochastic error.” In this paper we use the same methodology.

The pure “Downsian” spatial model of voting tends to predict that parties will converge to the center of the electoral distribution [10–12]. However, when valence is included, the prediction is very different. To see this suppose there are two parties, A and B, and both choose the same position at the electoral center, but A has much higher valence than B. This higher valence indicates that voters have a bias towards party A and as a consequence more voters will choose A over B. The question for B is whether it can gain votes by moving away from the center. It should be obvious that the optimal position of both A and B will depend on the various estimated parameters of the model. To answer this question we now present the details of the spatial model.

### 2.1. The Theoretical Model

To find the optimal party positions to the anticipated electoral outcome we use a Downsian vote model that has a valence component as presented in Schofield [9]. Let the set of parties be denoted by *P* = 1,…, *p*. The positions of the *p* parties (We will use candidate, party and agents interchangeably throughout the paper.) in *X*⊆ℝ^*w*^ where *w* is the dimension of the policy space it is given by the vector

(1) $$\begin{array}{c} z=\left(z_{1},\ldots ,z_{j},\ldots ,z_{p}\right)\in X^{p}. \end{array}$$

Denote voter *i*'s ideal policy be *x*_*i*_ ∈ *X* and her utility by *u*_*i*_(*x*_*i*_, *z*) = (*u*_*i*1_(*x*_*i*_, *z*_1_),…, *u*_*ip*_(*x*_*i*_, *z*_*p*_)), where

(2) $$\begin{array}{c} u_{ij}\left(x_{i},z_{j}\right)=\lambda_{j}-\beta {||x_{i}-z_{j}||}^{2}+\epsilon_{j}=u_{ij}^{*}\left(x_{i},z_{j}\right)+\epsilon_{j}. \end{array}$$

Here *u*_ij_*(*x*_*i*_, *z*_*j*_) is the observable component of the utility voter *i* derived from party *j*. The competence valence of candidate *j* is *λ*_*j*_, and the competence valence vector ***λ*** = (*λ*_1_, *λ*_2_,…, *λ*_*p*_) is such that *λ*_*p*_ ≥ *λ*_*p*−1_ ≥ ⋯≥*λ*_2_ ≥ *λ*_1_, so that party 1 has the lowest valence. Note that *λ*_*j*_ is the same for all voters and provides an estimate of the “quality” of party *j* or its ability to govern. The term ||*x*_*i*_ − *z*_*j*_|| is simply the Euclidean distance between voter *i*'s position *x*_*i*_ and candidate *j*'s position *z*_*j*_. The coefficient *β* is the weight given to this policy difference. The error vector **ϵ** = (*ϵ*_1_,…, *ϵ*_*j*_,…, *ϵ*_*p*_) has a Type I extreme value distribution, where the variance of *ϵ*_*j*_ is fixed at *π* ^2^/6. Note that *β* has dimension 1/*L* ^2^  , where *L* is whatever unit of measurement used in *X*.

Since voter behavior is modeled by a probability vector, the probability that voter *i* chooses party *j* when parties position themselves at **z** is

(3) ρij(z)=Pr[uij(xi,zj)>uil(xi,zl), ∀l≠j]=Pr[ϵl−ϵj<uij∗(xi,zj)−uil∗(xi,zj), ∀l≠j].

Here Pr stands for the probability operator generated by the distribution assumption on **ϵ**. Thus, the probability that *i* votes for *j* is given by the probability that *u*_*ij*_(*x*_*i*_, *z*_*j*_) > *u*_*ij*_(*x*_*i*_, *z*_*l*_) for all *l* ≠ *j* ∈ *P*, that is, that *i* gets a higher utility from *j* than from any other party.

Train [32] showed that when the error vector **ϵ** has a Type I extreme value distribution, the probability *ρ*_*ij*_(*z*) has a Multinomial Logit (MNL) specification and can be estimated. Thus, for each voter *i* and party *j*, the probability that voter *i* chooses party *j* at the vector **z** is given by

(4) $$\begin{array}{c} \rho_{ij}\left(z\right)=\frac{\exp\left[u_{ij}^{*}\left(x_{i},z_{j}\right)\right]}{\sum_{k=1}^{p}\exp u_{ik}^{*}\left(x_{i},z_{k}\right)}. \end{array}$$

Voters decisions are stochastic in this framework. (See, for example, the models of McKelvey and Patty [14]. Note that there is a problem with the independence of irrelevant alternatives assumption (IIA) which can be avoided using a probit model [33]. However, Quinn et al. [34] have shown that probit and logit models tend to give very similar results. Indeed the results given here for the logit model carry through for probit, though they are less elegant.) Even though parties cannot perfectly anticipate how voters will vote, they can estimate the *expected* vote share of party *j* as the average of these probabilities as follows:

(5) $$\begin{array}{c} V_{j}\left(z\right)=\frac{1}{n}\sum_{i\in N}\rho_{ij}\left(z\right). \end{array}$$

We assume a party's objective is to find the position that maximizes its expected vote share, as desired by “Downsian” opportunists. On the other hand, the party may desire to adopt a position that is preferred by the base of the party supporters, namely, the “guardians” of the party, as suggested by Roemer [35].

We assume that parties can estimate how their vote shares would change if they *marginally* move their policy position. The Local Nash Equilibrium (LNE) is that vector **z** of party positions such that no party may shift position by a small amount to increase its vote share. More formally a LNE is a vector **z** = (*z*_1_,…, *z*_*j*_,…, *z*_*p*_) such that each vote share *V*_*j*_(**z**) is weakly locally maximized at the position *z*_*j*_. To avoid problems with zero eigenvalues we also define a SLNE to be a vector that *strictly* locally maximizes *V*_*j*_(**z**).

Using the estimated MNL coefficients we simulate these models and then relate any vector of party positions, **z**, to a vector of vote share functions *V*(**z**) = (*V*_1_(**z**),…, *V*_*p*_(**z**)), predicted by the particular model with *p* parties. Moreover, we can examine whether in equilibrium parties position themselves at the electoral mean. (The electoral mean or origin is the mean of all voters' positions, (1/*n*)∑*x*_*i*_ normalized to zero, so that (1/*n*)∑*x*_*i*_ = 0.) We call this vector *the electoral mean*.

Given the vector of policy position **z**, and since the probability that voter *i* votes for party *j* is given by (4), the impact of a *marginal* change in *j*'s position on the probability that *i* votes for *j* is then

(6) $$\begin{array}{c} {\frac{d\rho_{ij}\left(z\right)}{dz_{j}}|}_{z_{-j}}=2\beta \rho_{ij}\left(1-\rho_{ij}\right)\left(x_{i}-z_{j}\right), \end{array}$$

where **z**_−*j*_ indicates that we are holding the positions of all parties but *j* is fixed. The effect that *j*'s change in position has on the probability that *i* votes for *j* depends on the weight given to the policy differences with parties, *β*; on how likely is *i* to vote for *j*, *ρ*_*ij*_, and for any other party, (1 − *ρ*_*ij*_) and on how far apart *i*'s ideal policy is from *j*'s, (*x*_*i*_ − *z*_*j*_).

From (5), party *j* adjusts its position to maximize its expected vote share, that is, *j*'s first order condition is

(7) $$\begin{array}{c} {\frac{dV_{j}\left(z\right)}{dz_{j}}|}_{z_{-j}}=\frac{1}{n}\sum_{i\in N}\frac{d\rho_{ij}}{dz_{j}}=\frac{1}{n}\sum_{i\in N}2\beta \rho_{ij}\left(1-\rho_{ij}\right)\left(x_{i}-z_{j}\right)=0, \end{array}$$

where the third term follows after substituting in (6). The FOC for party *j* in (7) is satisfied when

(8) $$\begin{array}{c} \sum_{i\in N}^{}\rho_{ij}\left(1-\rho_{ij}\right)\left(x_{i}-z_{j}\right)=0 \end{array}$$

so that the *candidate* for party *j*'s vote maximizing policy (See Schofield [36] for the proof.) is

(9) $$\begin{array}{c} z_{j}^{C}=\sum_{i\in N}\alpha_{ij}x_{i},\;\text{where}\;\;\alpha_{ij}\equiv \frac{\rho_{ij}\left(1-\rho_{ij}\right)}{\sum_{i\in N}\rho_{ij}\left(1-\rho_{ij}\right)}, \end{array}$$

where *α*_*ij*_ represents the weight that party *j* gives to voter *i* when choosing its candidate vote maximizing policy. This weight depends on how likely is *i* to vote for *j*, *ρ*_*ij*_, and for any other party, (1 − *ρ*_*ij*_) relative to all voters. (For example, if all voters are equally likely to vote for *j*, say with probability *v*, then the weight party *j* gives to voter *i* in its vote maximizing policy is 1/*n*; that is, the weight *j* gives each voter is just the inverse of the population size.) Note that *α*_*ij*_ may be nonmonotonic in *ρ*_*ij*_. To see this exclude voter *i* from the denominator of *α*_*ij*_. When ∑_*a*∈*N*−*i*_ *ρ*_*aj*_(1 − *ρ*_*aj*_) < 2/3 then *α*_*ij*_  (*ρ*_*ij*_ = 0) < *α*_*ij*_ (*ρ*_*ij*_ = 1) < *α*_*ij*_ (*ρ*_*ij*_ = 1/2). Thus, if *i* will for sure vote for *j*, *i* receives a lower weight in *j*'s candidate position than a voter who will only vote for *j* with probability 1/2 (an “undecided” voter). Party *j* caters then to “undecided” voters by giving them a higher weight in *j*'s policy weight and thus a higher weight on its position. This is the most common case. When ∑_*a*∈*N*−*i*_ *ρ*_*aj*_(1 − *ρ*_*aj*_) > 2/3, then *α*_*ij*_ increases in *ρ*_*ij*_. If *j* expects a large enough vote share (excluding voter *i*), it gives a core supporter (a voter who votes for sure for *j*) a higher weight in its policy position than it gives other voters as there is no risk of doing so. The weights *α*_*ij*_ are endogenously determined in the model.

Note that since voter *i*'s utility depends on how far *i* is from party *j*, the probability that *i* votes for *j* given in (4) and the expected vote share of the party given in (5) are influenced by the voters and parties positions in the policy space. That is, in the empirical models estimated below, the positions of voters and parties in the policy space, together with the valence estimates, influence voters electoral choices.

Recall that we are interested in finding whether parties converge to or diverge from the electoral mean. Suppose that *all* parties locate at the same position, *z*_*k*_ = *z* for all *k* ∈ *P*. Thus, from (2) we see that

(10) $$\begin{array}{c} \left[u_{ik}^{*}\left(x_{i},z\right)-u_{ij}^{*}\left(x_{i},z\right)\right]=\left(\lambda_{k}-\lambda_{j}\right), \end{array}$$

so the probability that *i* votes for *j* in (4) is given by

(11) ρij(z)=1∑k=1pexp⁡[uik∗(xi,zk)−uij∗(xi,zj)]=[∑k=1pexp⁡(λk−λj)]−1.

Clearly, in this case, *ρ*_*ij*_(**z**) = *ρ*_*j*_(**z**) is independent of voter *i*'s ideal point. Thus, from (9), the weight given by *j* to each voter is also independent of voter *i*'s position and given by

(12) $$\begin{array}{c} \alpha_{j}\equiv \frac{\rho_{j}\left(1-\rho_{j}\right)}{\sum_{i\in N}\rho_{j}\left(1-\rho_{j}\right)}=\frac{1}{n}, \end{array}$$

so that *j* gives each voter equal weight in its policy position. In this case, from (9), *j*'s candidate position is

(13) $$\begin{array}{c} z_{j}^{C}=\frac{1}{n}\sum_{i\in N}x_{i}, \end{array}$$

that is, *j*'s candidate position is to locate at the electoral mean which we have placed at the electoral origin. Let **z**_0_ = (0,…, 0) be the vector of party positions when all parties are at the electoral mean.

Moreover, as (11) indicates when parties locate at the mean **z**_0_, only valence differences between parties matter in voters' choices. The probability that a generic voter votes for party 1 (the party with the lowest valence) is

(14) $$\begin{array}{c} \rho_{1}\equiv \rho_{1}\left(z_{0}\right)={\left[\sum_{k=1}^{p}\exp\left(\lambda_{k}-\lambda_{1}\right)\right]}^{-1}. \end{array}$$

Using this spatial model, Schofield [9] proved a *Valence Theorem* determining whether vote maximizing parties locate at the mean. The theorem showed that the spatial model is characterized by a *convergence coefficient* given by

(15) $$\begin{array}{c} c\equiv c\left(\lambda ,\beta ,\sigma^{2}\right)=2\beta \left[1-2\rho_{1}\right]\sigma^{2}. \end{array}$$

The convergence coefficient depends on *β*, the weight given to policy differences; on *ρ*_1_, the probability that a generic voter votes for the lowest valence party at the vector **z**_0_ and on *σ* ^2^, the *electoral variance* given by

(16) $$\begin{array}{c} \sigma^{2}\equiv \text{trace}\left(\nabla \right), \end{array}$$

where ∇ is the symmetric *w* × *welectoral covariance matrix*. (∇ is simply a description of the distribution of voter preferred points taken about the electoral mean.)

The convergence coefficient increases in *β* and *σ* ^2^ (and on its product *βσ* ^2^) and decreases in *ρ*_1_. As (14) indicates *ρ*_1_ decreases if the valence differences between party 1 and the other parties increases, that is, when the difference between *λ*_1_ and {*λ*_2_,…, *λ*_*p*_} increases.

The Valence Theorem allows us to characterize polities according to the value of their convergence coefficient. The theorem states that when the *sufficient* condition for convergence to the electoral mean is met, that is, when *c* < 1, the LNE is one where all parties adopt the same position at the mean of the electoral distribution. A *necessary* condition, for convergence to the electoral mean is that *c* < *w*, where *w* is the dimension of the policy space. If *c* ≥ *w*, then there may exist a nonconvergent LNE. Note that in this case, there may indeed be no LNE. However, there will exist a mixed strategy Nash equilibrium (MNE). In either of these two cases we expect at least one party will *diverge* from the electoral mean.

Note that *c* is dimensionless, because *βσ* ^2^ has no dimension. In a sense *βσ* ^2^ is a measure of the polarization of the preferences of the electorate. Moreover, *ρ*_1_ in (14) is a function of the distribution of beliefs about the competence of party leaders, which is a function of the difference (*λ*_*k*_ − *λ*_1_).

When some parties have a low valence, so the probability that a generic voter votes for party 1 (with the lowest valence when all parties locate at the origin), *ρ*_1_ in (14) will tend to be small because the valence differences between party 1 and the other parties is *sufficiently large*. Thus, vote maximizing parties will *not all* converge to the electoral mean. In this case *c* will be close to 2*βσ* ^2^. If 2*βσ* ^2^ is large because, for example, the electoral variance is large, then *c* will be large, suggesting *c* > *w*. In this case, the low valence party has an incentive to move away from the origin to increase its vote share. This implies the existence of a *centrifugal* force pulling some parties away from the origin.

Thus, for *βσ* ^2^ sufficiently large so that *c* ≥ *w*, we expect parties to diverge from the electoral center. Indeed, we expect those parties that exhibit the lowest valence to move further away from the electoral center, implying that the centrifugal force on parties will be significant. Thus, in fragmented polities with a polarized electorate, the nature of the equilibrium tends to maintain this *centrifugal* characteristic.

On the contrary, in a polity where there are no very small or low valence parties, *ρ*_1_ will tend to 1/2 and so *c* will be small. In a polity with small *βσ* ^2^ and with low valence differences, so that *c* < 1, we expect all parties to converge to the center. In this case, we expect this *centripetal* tendency to be maintained.

The convergence coefficient is a way of characterizing the Hessian (the *w* by *w* *second* derivatives of the vote share function) of party 1 with the lowest valence. The Hessian of the vote share function of party 1 is given by the characteristic matrix

(17) $$\begin{array}{c} C_{1}=2\beta \left(1-2\rho_{1}\right)\nabla -I. \end{array}$$

Here *I* is a *w* by *w* identity matrix and the other terms are as before. The eigenvalues of *C*_1_ determine whether the vote share function of party 1 will be at a maximum, minimum, or at a saddlepoint at the electoral mean. If *C*_1_ shows that party 1 is at a minimum or at a saddlepoint at the mean then party 1 has an incentive to locate away from the mean to increase its vote share. When all parties are at the mean and *c* < 1, then all eigenvalues of the Hessian of the vote share function of the lowest valence party are negative indicating that the vote share function is at a maximum. The LNE must then be at the electoral mean.

For an arbitrary dimension, *w*, if *c*(***λ***, *β*, *σ* ^2^) ≤ 1 in (15), then trace (*C*_1_) < 0. In the two-dimensional case, if *c*(***λ***, *β*, *σ* ^2^) < 1, then det⁡ (*C*_1_) must be positive, implying that both eigenvalues of *C*_1_ are negative. It then follows that all {*C*_*j*_} have negative eigenvalues, giving a SLNE and thus an LNE at the electoral mean. (This result follows from the application of the triangle inequality to the determinant. A parallel result can be obtained in more than two dimensions.)

The Valence Theorem asserts that if *c*(***λ***, *β*, *σ* ^2^) > *w* then the party with the lowest valence has an incentive to move away from the electoral mean to increase its vote share. When this is the case then other low valence parties may also find it advantageous to vacate the center. The value of the convergence coefficient, together with the analysis of the Hessians of the low valence parties, allows us to identify which parties have an incentive to move away from the electoral mean. The convergence coefficient then gives an easy and intuitive way to identify whether a low valence party should vacate the electoral mean.

In the next section, we estimate the convergence coefficient of various elections in different countries.

## 3. MNL Models of the Elections of Various Countries

We use the framework of the spatial model presented in Section 2 as a unifying methodology that allows us to study convergence across elections, countries, and political regimes. The Valence Theorem leads to the convergence coefficient of the election, a summary statistic that determines whether parties converge to or diverge from the electoral mean. Using this formal multinomial (MNL) spatial model, we now estimate the convergence coefficient for the elections in various countries. For each MNL estimation we choose a baseline party and normalize its coefficients to zero, then estimate the coefficients of all other parties relative to those of the base party. Using these coefficients we estimate the convergence coefficient and the characteristic matrix of the low valence parties to determine whether these parties converge to or diverge from the electoral mean in each election for each country. (These elections were studied in depth elsewhere. In this paper, we present only the calculations leading to the convergence coefficient and estimate the confidence intervals for the convergence coefficients that were not provided in earlier work.)

We study convergence under three political regimes (plurality, proportional representation, and anocracy) and group countries according to the similarities of their political regimes. Under plurality rule, we examine elections in two Anglo-Saxon countries: the US and the UK; under proportional representation we study Israel, Turkey, and Poland; and under anocracy, Georgia, Russia, and Azerbaijan. Since we use the same unifying methodology for all countries we present the methodology for the first elections in detail then condense the analysis to its basic components for the remaining countries. For each country we give a general description of the analysis and direct the reader to the full analysis of each election in the detailed country paper. We summarize the results across countries in various tables.

### 3.1. Convergence in Plurality Systems

We begin our analysis by examining the United States and the United Kingdom. Elections in these countries are carried out under plurality rule. We show that the electoral system in these countries produces relatively low convergence coefficients. (Relative to the convergence coefficient of other countries included in this study. In Section 4 we discuss how the values of the convergence coefficient are related to the political systems under which the countries operate.)

#### 3.1.1. The 2000, 2004, and 2008 Elections in the United States

**Figure 1:**
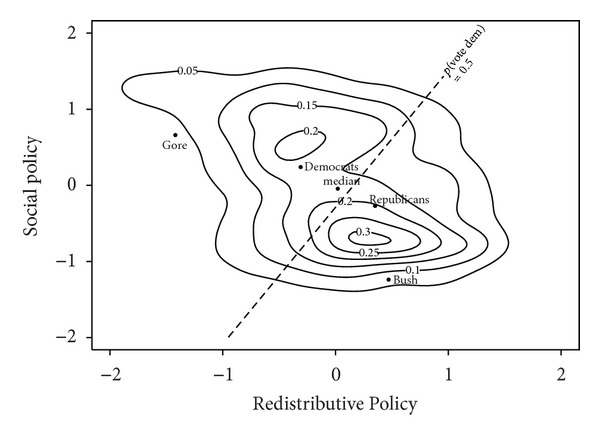
Distribution of voter ideal points and candidate positions in the 2000 US election.

**Figure 2:**
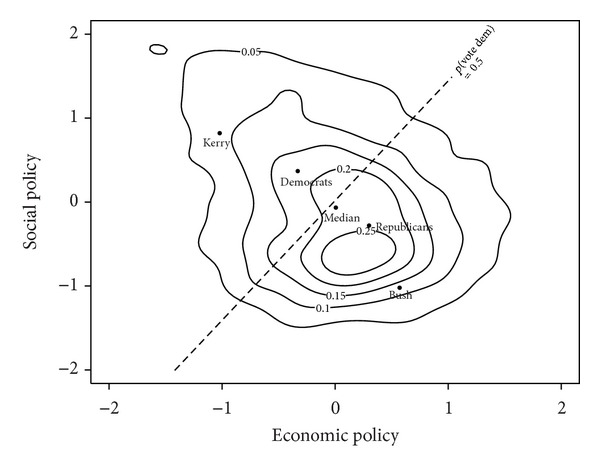
Distribution of voter ideal points and candidate positions in the 2004 US election.

**Figure 3:**
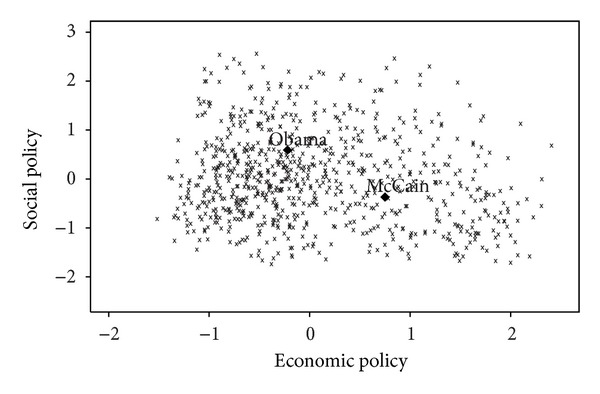
Distribution of voter ideal points and candidate positions in the 2008 US election.

We construct stochastic models of the 2000, 2004, and 2008 US presidential elections using survey data taken from the American National Election Surveys (ANES). The factor analysis done on ten survey questions taken from the ANES (See Schofield et al. [30, 31] for the list of survey questions and the factor loadings and the full analysis of the US elections.) led us to conclude that voters preferences can be represented along the economic (*E* = *x*-axis) and social (*S* = *y*-axis) dimensions for all three elections. Voters located on the left of the economic axis are pro-redistribution. The social axis is determined by attitudes to abortion and gays. We interpreted greater values along this axis to mean more support for certain civil rights. Using the factor loadings we estimated each voter's position in these two dimensions. Figures 1, 2, and 3 give a smoothing of the estimated voter distribution of the 2000, 2004, and 2008 elections, respectively.

Voters' ideal points in the *2000 US election* are characterized by the following *electoral covariance matrix*:

(18) $$\begin{array}{c} \nabla_{2000}^{\text{US}}=\left[\begin{array}{cc} \sigma_{E}^{2}=0.58 & \sigma_{ES}=-0.20\\ \sigma_{ES}=-0.20 & \sigma_{S}^{2}=0.59 \end{array}\right]. \end{array}$$

The trace of electoral covariance matrix is *σ*_US 2000_^2^ ≡ trace (∇_US_^2000^) = *σ*_*E*_^2^ + *σ*_*S*_^2^ = 1.17. Given the negative covariance between these two dimensions, *σ*_*ES*_ = −0.20, the correlation between these two factors is −0.344.

Using the spatial model presented in Section 2, we estimated the MNL model of the 2000 election. The coefficients for the US 2000 shown in Table 1 are

(19) $$\begin{array}{c} \lambda_{\text{rep}}^{\text{US}2000}=-0.43,\;\;\lambda_{\text{dem}}^{\text{US}2000}\equiv 0.0,\;\;\beta_{2000}^{\text{US}}=0.82. \end{array}$$

Bush's competence valence, *λ*_rep_^US2000^ = −0.43, measures the common perception that voters in the sample have on Bush's ability to govern and represents the nonpolicy component in the voter's utility function in (2). As seen in Table 1, for the 2000 election Bush has a statistically significant *lower* valence than Gore, the democratic (baseline) candidate. Bush's negative valence is an indication that voters regarded him as less able to govern than Gore, once policy differences are taken into account.

To find the convergence coefficient for this election, we assume that *all* parties locate at the electoral mean so that parties differ only in their valence terms (see Section 2). We can use (14) and the coefficients in (19) to estimate the probability that a typical US voter chooses to vote for the low valence Republican candidate, when both Bush and Gore locate at origin, **z**_0_; that is,

(20) ρrepUS2000=[∑k=12exp⁡(λkUS2000−λrepUS2000)]−1=[1+exp⁡(0.43)]−1=0.40.

We found the estimate for *ρ*_rep_^US2000^ using the MNL valence estimates. Note that since the central estimates of ***λ*** = (*λ*_1_,…, *λ*_*p*_) given by the MNL regressions depend on the sample of voters surveyed then so does *ρ*_1_. Thus, to make inferences from empirical models we need the 95% confidence bounds of *ρ*_1_. In the introduction of the appendix we derive the methodology used to find the confidence bounds of *ρ*_1_. The bounds of *ρ*_1_ are calculated in Appendix A.1.

The results indicate that in the 2000 election, both candidates found it in their best interest to locate at the electoral mean. To see this, we compute the convergence coefficient using (15) and the electoral covariance matrix in (18) ∇_US_^2000^ to determine whether the two parties converge to, or diverge from, the electoral mean.

Using (19) and (20) we have that 2*β*_2000_^US^(1 − 2*ρ*_rep_^US2000^) = 2 × 0.82 × 0.2 = 0.328 and from (18) the trace is *σ*_US2000_^2^ = 1.17 so that using (15) the convergence coefficient for 2000 US election is

(21) $$\begin{array}{c} c_{\text{US}}^{2000}\equiv 2\beta_{2000}^{\text{US}}\left(1-2\rho_{\text{rep}}^{\text{US}2000}\right)\sigma_{\text{US}2000}^{2}=0.328\times 1.17=0.384. \end{array}$$

Appendix A.1 shows that *c*_US_^2000^ is significantly less than 1 implying that *c*_US_^2000^ meets the sufficient and thus necessary condition for convergence to the electoral mean given in Section 2.

To check whether Bush, the low valence candidate, has an incentive to stay at the electoral origin, **z**_0_, that is, whether Bush's vote share function is at a maximum at **z**_0_, we use the Hessian or characteristic matrix (of *second* order conditions) of Bush's vote share function using (17) at **z**_0_ as follows:

(22) CrepUS2000=[2β2000US(1−2ρrepUS2000)]∇2000US−I=0.328[0.58−0.20−0.200.59]−I=[−0.81−0.06−0.06−0.81].

Because the characteristic matrix for Bush *C*_rep_^US2000^ is estimated using the MNL coefficients of the 2000 US sample, *C*_rep_^US2000^ depends on the sample of voters surveyed. The confidence bounds on *C*_rep_^US2000^ in Appendix A.1 suggest that if Bush positions himself at the electoral origin, then with probability exceeding 95%, his vote share function would be at a maximum. We infer that, with probability exceeding 95%, the origin is an LNE for the spatial model for the 2000 US election. The valence differences between Bush and Gore are not large enough to cause either of them to move from the origin. The unique local Nash equilibrium was one where both candidates converge to the electoral origin in order to maximize their vote shares.

All the components needed to derive the convergence coefficient for 2000 US election and its confidence bounds are summarized in Table 2.

Bush faced Kerry as the democratic candidate in the *2004 US election*. The distribution of voters in 2004 gives the following electoral covariance matrix along the economic and social dimensions:

(23) $$\begin{array}{c} \nabla_{2004}^{\text{US}}=\left[\begin{array}{cc} \sigma_{E}^{2}=0.58 & \sigma_{ES}=-0.177\\ \sigma_{ES}=-0.177 & \sigma_{S}^{2}=0.59 \end{array}\right]. \end{array}$$

While the covariance between economic and social axes differs, the trace *σ*_US2004_^2^ = trace (∇_US_^2004^) = *σ*_*E*_^2^ + *σ*_*S*_^2^ = 1.17 is similar to that in the 2000 US election.

From Table 1, the MNL estimates of the spatial model for the 2004 US election are

(24) $$\begin{array}{c} \lambda_{\text{rep}}^{\text{US}2004}=-0.43,\;\;\lambda_{\text{dem}}^{\text{US}2004}\equiv 0.0,\;\;\beta_{2004}^{\text{US}}=0.95. \end{array}$$

Bush has a significantly lower valence (*λ*_rep_^US2004^ = −0.43) than Kerry (*λ*_dem_^US2004^ ≡ 0.0), the baseline candidate.

From (14) the probability that a US voter chooses Bush, the low valence candidate, when both Bush and Kerry are at the electoral origin, **z**_0_, is

(25) ρrepUS2004=[∑k=12exp⁡(λkUS2004−λrepUS2004)]−1=[1+exp⁡(0.43)]−1=0.40.

The confidence bounds for *ρ*_rep_^US2004^ are given in Appendix A.1. Since Bush's valence, relative to that of his opponent, was similar in the two elections, it is not surprising that the probability of voting Republican is similar in the two elections, compare (20) and (25). From (15), 2*β*_2004_^US^(1 − 2*ρ*_rep_^US2004^) = 2 × 0.95 × 0.2 = 0.38 and *σ*_US2004_^2^ = 1.17, so that the convergence coefficient of the 2004 election is

(26) $$\begin{array}{c} c_{\text{US}}^{2004}=2\beta_{2004}^{\text{US}}\left[1-2\rho_{\text{rep}}^{\text{US}2004}\right]\sigma_{\text{US}2004}^{2}=0.38\times 1.19=0.45. \end{array}$$

Since *c*_US_^2004^ = 0.45 is significantly less than 1 (see Appendix A.1), the sufficient condition for convergence given in Section 2 is met. Moreover, from (17) Bush's characteristic matrix is

(27) CrepUS2004=[2β2004US(1−2ρrepUS2004)]∇2004US−I=0.38[0.53−0.18−0.180.66]−I=[−0.80−0.06−0.06−0.75].

If Bush positions himself at the electoral origin, then with probability exceeding 95% (see Appendix A.1), his vote share function would be at a maximum. Bush, the low valence candidate, has then no incentive to move from the origin, **z**_0_. With probability exceeding 95%, the mean is an LNE for model of the 2004 US election.

Our analysis suggests that Obama's victory over McCain in the *2008 US election* was the result of an overall shift in the relative valences of the Democratic and Republican candidates as compared to those of the candidates in the 2000 and 2004 elections. The electoral covariance matrix for the sample in 2008 along the economic and social dimensions is

(28) $$\begin{array}{c} \nabla_{2008}^{\text{US}}=\left[\begin{array}{cc} \sigma_{E}^{2}=0.80 & \sigma_{ES}=-0.127\\ \sigma_{ES}=-0.127 & \sigma_{S}^{2}=0.83 \end{array}\right]. \end{array}$$

Relative to the two previous elections the “variance” of the electoral distribution *σ*_US2008_^2^ = trace (∇_2008_^US^) = *σ*_*E*_^2^ + *σ*_*S*_^2^ = 1.63 increased, while the covariance between these dimensions *σ*_*ES*_ = −0.127 decreased.

The MNL estimates of the spatial model given in Table 1 for the 2008 US election are

(29) $$\begin{array}{c} \lambda_{\text{rep}}^{\text{US}2008}=-0.84,\;\;\lambda_{\text{dem}}^{\text{US}2008}\equiv 0.0,\;\;\beta_{2008}^{\text{US}}=0.85. \end{array}$$

Obama, the baseline candidate, has a significantly higher valence than McCain.

From (14) the probability that a voter chooses McCain, when both candidates are at the origin, **z**_0_, is

(30) ρrepUS2008=[∑k=12exp⁡(λkUS2008−λrepUS2008)]−1=[1+exp⁡(0.84)]−1=0.30.

From (15), 2*β*_US_^2008^(1 − 2*ρ*_dem_^US2008^) = 2 × 0.85 × 0.4 = 0.68, and *σ*_US2008_^2^ = 1.63, so the convergence coefficient is

(31) cUS2008=2β2008US[1−2ρdemUS2008]σUS20082=0.68×1.63=1.11.

Appendix A.1 shows that *c*_US_^2008^ = 1.11 is significantly greater than 1 and significantly less than 2. The Valence Theorem then states that the necessary but not the sufficient condition for convergence has been met. To check whether the low valence candidate, McCain, has an incentive to move from the electoral mean, we examine McCain's characteristic matrix using (17) to get

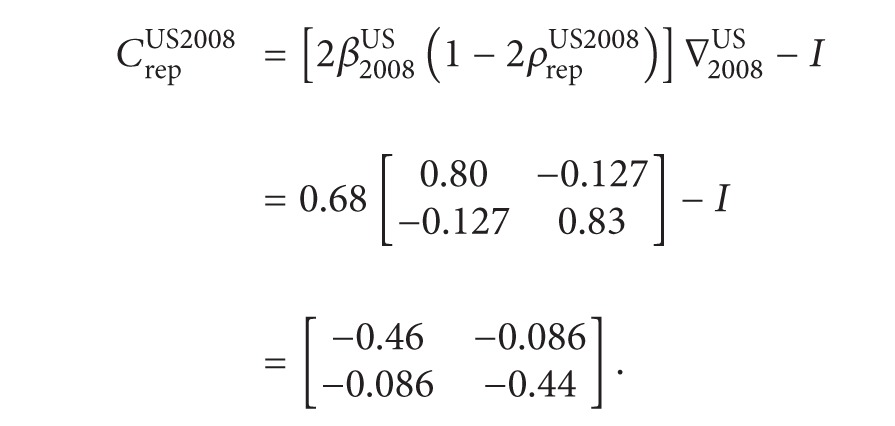
(32)
With probability exceeding 95% (see Appendix A.1) McCain's vote share function is at a maximum when he locates at the origin, and thus has no incentive to move. Thus, with probability exceeding 95%, the electoral origin is an LNE for the spatial model for the 2008 US election.

In conclusion, Table 2 illustrates that the convergence coefficient varies across elections in the same country even when there are only two parties. This is to be expected as from (15) the convergence coefficient depends on the “variance” of the electoral distribution, *σ* ^2^ = trace(∇); on the weight voters give to differences with party's policies, *β*; and on the probability that a voter chooses the party with the lowest valence, *ρ*_1_. The electoral distributions of the 2000 and 2004 are quite similar, as can be seen by comparing (18) and (23). Voters' preferences had however substantially changed by 2008, see (28). The electoral variance along both axes increased relative to 2000 and 2004. While the 2000 and 2004 convergence coefficients are indistinguishable from each other, the 2008 coefficient is significantly different from that in 2000 and 2004. In spite of these differences, candidates in all three elections had no incentive to move from the origin.

#### 3.1.2. The 2005 and 2010 Elections in Great Britain

**Figure 4:**
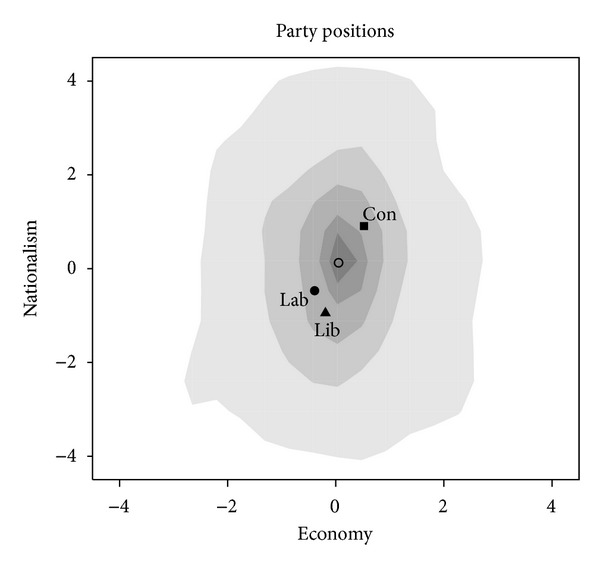
Electoral distribution and estimated party positions in Britain in 2005.

**Figure 5:**
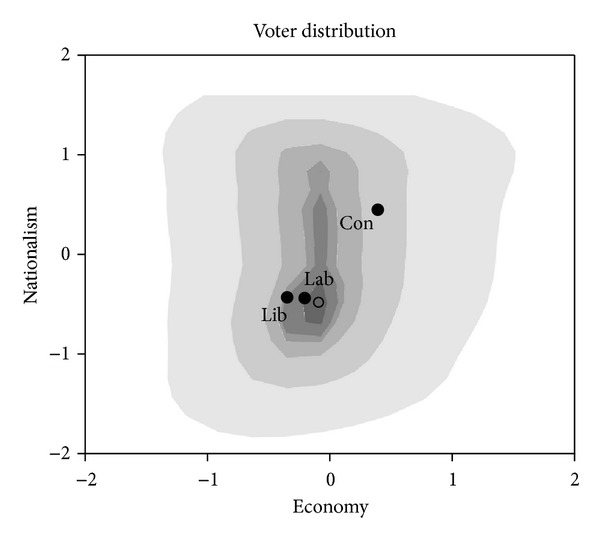
Voter and party positions in Britain in 2010.

We study the 2005 and 2010 elections in the UK using the British Election Study (BES). (The full analysis of the 2005 and 2010 elections in Great Britain can be found in Schofield et al. [37].) The factor analysis conducted on the questions of the two surveys led us to conclude that the same two dimensions mattered in voter choices in the two elections. The first factor deals with issues on “EU membership,” “Immigrants,” “Asylum seekers,” and “Terrorism.” A voter who feels strongly about nationalism has a high value in the *nationalism* dimension (Nat = *x*-axis). Items such as “tax/spend,” “free market,” “international monetary transfer,” “international companies,” and “worry about job loss overseas” have strong influence in the *economic* (*E* = *y*-axis) dimension with higher values indicating a promarket attitude. Figures 4 and 5 present the smoothed electoral distribution obtained from these analyses for the 2005 and 2010 elections.

The electoral covariance matrix for the *2005 UK election* is

(33) $$\begin{array}{c} \nabla_{2005}^{\text{UK}}=\left[\begin{array}{cc} \sigma_{\text{Nat}}^{2}=1.646 & \sigma_{\text{Nat}E}=0.00\\ \sigma_{E\text{Nat}}=0.067 & \sigma_{E}^{2}=3.961 \end{array}\right], \end{array}$$

where *σ*_UK2005_^2^ ≡ trace(∇_2005_^UK^) = *σ*_Nat_^2^ + *σ*_*E*_^2^ = 5.607.

From Table 1, the MNL estimates of the spatial model for the 2005 UK are

(34) λLabUK2005=0.52,    λConUK2005=0.27,λLibUK2005≡0.0,  β2005UK=0.15.

Both the Labour (Lab) and the Conservative (Con) parties had a significantly higher valence than the Liberal Democrats (Lib), the baseline party.

From (14), the probability that a voter chooses the Liberal Democratic Party, the lowest valence party, when all parties locate at the origin, **z**_0_, is

(35) ρLibUK2005=[∑k=13exp⁡(λkUK2005−λLibUK2005)]−1=[1+exp⁡(0.52)+exp⁡(0.27)]−1=0.25.

Given that 2*β*_2005_^UK^(1 − 2*ρ*_Lib_^UK2005^) = 2 × 0.15 × 0.5 = 0.15 and since *σ*_UK2005_^2^ = 5.607 in (33), from (15) the convergence coefficient, in Table 2, is

(36) cUK2005=2β2005UK[1−2ρLibUK2005]σUK20052=0.15×5.607=0.84.

Appendix A.1 shows that *c*_UK_^2005^ is significantly less than 1 and thus meets the sufficient and necessary conditions for convergence given in Section 2. From (17) the characteristic matrix of the Liberal Democratic Party is

(37) CLib2005UK=[2β2005UK(1−2ρLibUK2005)]∇2005UK−I=0.15[1.6460.00.0673.961]−I=[−0.750.00.01−0.406].

From the 95% confidence bounds in Appendix A.1, we conclude that if the LibDem locates at the origin, it is maximizing its vote share and has no incentive to vacate the center. Thus, with probability exceeding 95%, the origin is an LNE for the 2005 UK election.

The electoral covariance matrix for the *2010 UK election* is

(38) $$\begin{array}{c} \nabla_{2010}^{\text{UK}}=\left[\begin{array}{cc} \sigma_{\text{Nat}}^{2}=0.601 & \sigma_{\text{Nat}E}=0.067\\ \sigma_{E\text{Nat}}=0.067 & \sigma_{E}^{2}=0.861 \end{array}\right], \end{array}$$

where *σ*_UK2010_^2^ ≡ trace(∇_2010_^UK^) = 1.462, lower than in 2005.

From Table 1, the MNL estimates of the spatial model of the 2010 election are

(39) λLabUK2010=−0.04,  λConUK2010=0.17,λLibUK2010≡0.0,  β2010UK=0.86.

Given the great popular discontent with Gordon Brown, the Labour leader, heading into the 2010 election, it is not surprising to find that both Conservatives and Liberal Democrats (the base party) had significantly higher valences than Labour.

From (14) the probability that a voter chooses Labour, when all parties locate at the origin, **z**_0_, is

(40) ρLabUK2010=[∑k=13exp⁡(λkUK2010−λLabUK2010)]−1=[1+exp⁡(0.21)+exp⁡(0.04)]−1=0.319.

Since 2*β*_2010_^UK^(1 − 2*ρ*_Lab_^UK2010^) = 2 × 0.86 × 0.362 = 0.622 and *σ*_UK2010_^2^ = 1.462 in (38), from (15) the convergence coefficient, in Table 2, is

(41) cUK2010=2β2010UK[1−2ρLab2010]σUK20102=0.622×1.462=0.91.

The convergence coefficient *c*_UK_^2010^ = 0.91 is significantly less than 1 (see Appendix A.1), meeting the sufficient and thus necessary condition for convergence. From (17), Labour's characteristic matrix is

(42) CLabUK2010=[2β2010UK(1−2ρLabUK2010)]∇2010UK−I=0.622[0.6010.0670.0670.861]−I=[−0.630.0420.042−0.46].

If Labour, the low valence party, locates at the origin, then with probability exceeding 95%, its vote share function is at a maximum (see Appendix A.1) giving it no incentive to move from the mean. Thus, with probability exceeding 95%, the electoral origin is an LNE for the 2010 UK election.

The major shift in voters' preferences between the two elections led to very different electoral outcomes as evidenced by the electoral covariance matrices in (33) and (38). Voter dissatisfaction with the governing Labour leader led to a dramatic decrease in his competence valence and on the probability of voting Labour. Even though the electoral variance fell in 2010 relative to 2005, the increase in the convergence coefficient meant that this lower variance was more than compensated by the lower probability of voting Labour in 2010. The analysis for the UK elections shows that the convergence coefficient reflects not only changes in the electoral distribution but also changes in voters' valence preferences as the convergence coefficient of the 2005 election is substantially lower than the one for the 2010 election.

The analysis of these two Anglo-Saxon countries illustrate that even under plurality rule the convergence coefficient varies from election to election and from country to country. The analysis for the 2010 UK election highlights that candidates' valences matter and that parties understand how their valence affects their electoral prospects and may adjust their positions to increase their votes. This section illustrates that under plurality, the convergence coefficient has low values that generally satisfy the necessary condition for convergence to the mean and is thus below the dimension of the policy space.

### 3.2. Convergence in Proportional Systems?

We now estimate the convergence coefficients for three parliamentary countries using proportional representation: Israel, Turkey, and Poland. As is well known, these countries are characterized by multiparty elections in which generally no party wins a legislative majority leading then to coalitions governments. This section shows that these countries are characterized by very high convergence coefficients.

#### 3.2.1. The 1996 Election in Israel

In the 1996, as in previous elections, Israel had approximately nineteen parties attaining seats in the Knesset. (These include parties on the left, on the center, on the right, as well as religious parties. On the left there is Labor, Merets, Democrat, Communists and Balad; those on the center include Olim, Third Way, Center, Shinui; those on the right Likud, Gesher, Tsomet and Yisrael. The religious parties are Shas, Yahadut, NRP, Moledet, and Techiya.) There were small parties with 2 seats to moderately large parties such as Likud and Labor whose seat strengths lie in the range 19 to 44, out of a total of 120 Knesset seats. Since Likud and Labour compete for dominance of coalition government, these large parties must maximize their seat strength. Moreover, Israel uses a highly proportional electoral system with close correspondence between seat and vote shares. Thus one can consider vote shares as the maximand and for these parties.

**Figure 6:**
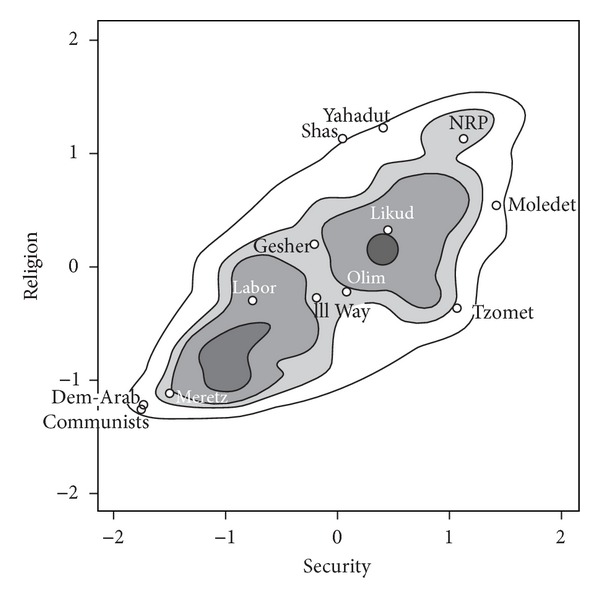
Party positions and voter distribution in Israel in the 1996 election.

Schofield et al. [30] performed a factor analysis of the surveys conducted by Arian and Shamir [38] for the 1996 Israeli election. The two dimensions identified by the factor analysis were Security (*S* = *x*-axis) and Religion (*R* = *y*-axis). “Security” refers to attitudes toward peace initiatives; “religion” to the significance of religious considerations in government policy. A voter on the left of the security axis is interpreted as supporting negotiations with the PLO, while higher values on the religious axis indicates support for the importance of the Jewish faith in Israel. The distribution of voters is shown in Figure 6.

Voter distribution along these two axes gives the following covariance matrix:

(43) $$\begin{array}{c} \nabla_{!996}^{\text{I}}=\left[\begin{array}{cc} \sigma_{S}^{2}=1.00 & \sigma_{SR}=0.591\\ \sigma_{RS}=0.591 & \sigma_{R}^{2}=0.732 \end{array}\right], \end{array}$$

giving a “variance” of *σ*_I1996_^2^ ≡ trace(∇_!996_^I^) = 1.732.

Only the seven largest parties are included in the MNL estimation. These include Likud, Labor, NRP, Moledat, Third Way (TW), and Shas with Meretz being the base party. From Table 2, the MNL coefficients for the 1996 election in Israel (I) are

(44) $$\begin{array}{c} \lambda_{\text{Lik}}^{\text{I}1996}=0.78,\;\;\lambda_{\text{Lab}}^{\text{I}1996}=0.999,\\ \lambda_{\text{NRP}}^{\text{I}1996}=-0.626,\;\;\lambda_{\text{MO}}^{\text{I}1996}=-1.259,\\ \lambda_{\text{TW}}^{\text{I}1996}\equiv -2.291,\;\;\lambda_{\text{Shas}}^{\text{I}1996}=-2.023,\\ \lambda_{\text{Merezt}}^{\text{I}1996}\equiv 0.0,\;\;\beta_{1996}^{\text{I}}=1.207. \end{array}$$

The *β*-coefficient and the valence estimates for all parties are significantly nonzero. The two largest parties, Likud and Labour, have significantly higher valences than the other smaller parties with Third Way (TW) having the smallest valence.

From (14), the probability that an Israeli votes for TW, when all parties locate at the mean is

(45) ρTWI1996=[∑k=17exp⁡[λjI1996−λTWI1996]]−1=[1+e3.071+e3.29+e1.665+ e1.032+e0.268+e2.291]−1≃0.014.

Given that 2*β*_1996_^I^(1 − 2*ρ*_TW_^I1996^) = 2 × 1.207 × 0.972 = 2.346 and since *σ*_I1996_^2^ = 1.732 from (43), then using (15) we compute the convergence coefficient for Israel, in Table 4, as

(46) c1996I=2β1996I(1−2ρTWI1996)σI19962=2.346×1.732=4.06.

The 95% confidence intervals for *c*_1996_^I^ = 4.06 in Appendix A.2 confirm that the necessary condition is *not* satisfied as *c*_1996_^I^ = 4.06 is significantly higher than 2, the dimension of the policy space. Moreover, at the electoral mean the vote share function of Third Way is *not* at a maximum since its Hessian from (17)

(47) CTWI1996=2β1996I(1−2ρTWI1996)∇!996I−I=2.346[1.000.5910.5910.732]−I=[1.3461.3861.3860.717]

shows that if TW locates at the mean its vote share function is at a saddlepoint since *C*_TW_^I1996^ has one positive (2.453) and one negative (−0.39) eigenvalue. Appendix A.2 confirms that *C*_TW_^I1996^ has one negative and one positive eigenvalue at both its lower and upper bounds. Thus, with a high degree of certainty TW deviates from the mean to maximize its votes and the electoral mean is *not* a LNE for the 1996 Israeli election.

#### 3.2.2. The 1999 and 2002 Elections in Turkey

**Figure 7:**
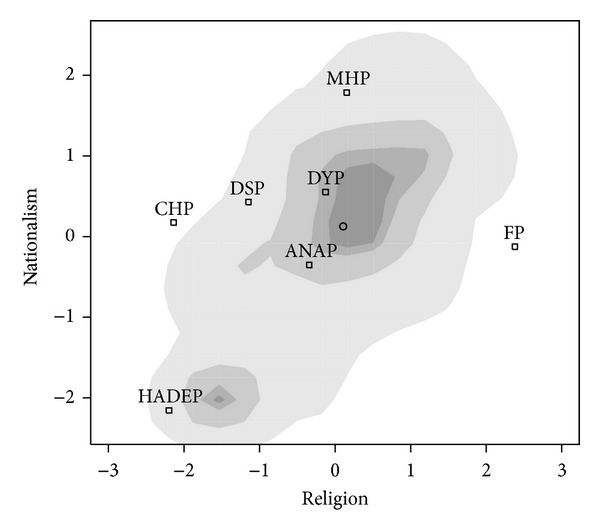
Party positions and voter distribution in the 1999 Turkish election.

**Figure 8:**
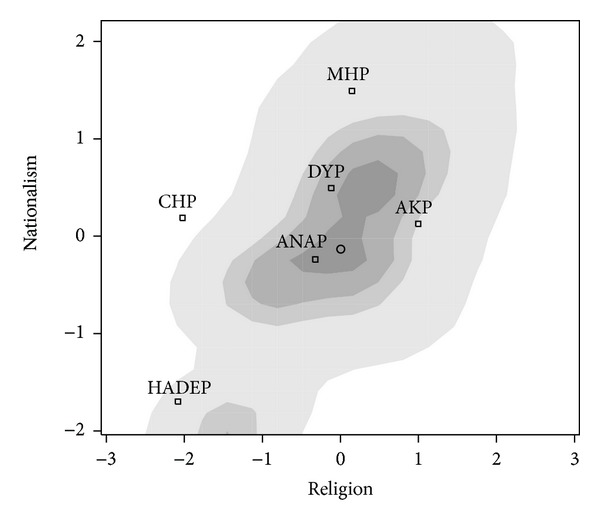
Party positions and voter distribution in Turkey in 2002.

We used factor analysis of electoral survey data of Veri Arastima for TUSES to study the 1999 and 2002 Turkish elections. (See Schofield et al. [39] for details of the estimation.) The analysis indicates that voters made decisions in a two-dimensional space during the two elections. Voters who support secularism or “Kemalism” are placed on the left of the Religious (*R* = *x*) axis and those supporting Turkish nationalism (*N* = *y*) to the north. Figures 7 and 8 give the distribution of voters along these two dimensions surveyed in these two elections.

**Figure 9:**
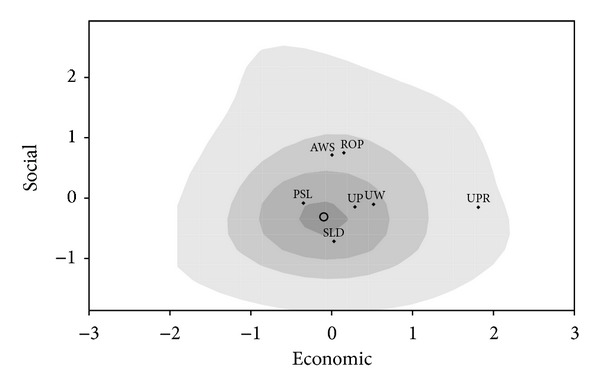
Voter distribution and party-positions in Poland in 1997.

Minor differences between these two figures include the disappearance of the Virtue Party (FP) which was banned by the Constitutional Court in 2001 and the change of the name of the pro-Kurdish party from HADEP to DEHAP. (For simplicity, the pro-Kurdish party is denoted HADEP in the various figures and tables. Notice that the HADEP position in Figures 8 and 9 is interpreted as secular and nonnationalistic.) The most important change is the emergence of the new Justice and Development Party (AKP) in 2002, essentially substituting for the outlawed Virtue Party.

The parties included in the analysis of the 1999 election are the Democratic Left Party (DSP), the National Action party (MHP), the Vitue Party (VP), the Motherland Party (ANAP), the True Path Party (DYP), the Republican People's Party (CHP), and the People's Democratic Party (HADEP). A DSP minority government formed, supported by ANAP and DYP. This only lasted about 4 months and was replaced by a DSP-ANAP-MHP coalition, indicating the difficulty of negotiating a coalition compromise across the disparate policy positions of the coalition members.

In *the 1999 election*, the electoral covariance matrix along the Religious (*R*) and Nationalism (*N*) axes is

(48) $$\begin{array}{c} \nabla_{!999}^{\text{T}}=\left[\begin{array}{cc} \sigma_{R}^{2}=1.20 & \sigma_{RN}=0.78\\ \sigma_{NR}=0.78 & \sigma_{N}^{2}=1.14 \end{array}\right], \end{array}$$

with *σ*_T1999_^2^ ≡ trace(∇_!999_^T^) = 2.34.

Using DYP as the base party, from Table 3, the 1999 MNL coefficients are

(49) $$\begin{array}{c} \lambda_{\text{FP}}^{\text{T}1999}=-0.16,\;\;\lambda_{\text{MHP}}^{\text{T}1999}=0.66,\\ \;\lambda_{\text{DYP}}^{\text{T}1999}\equiv 0.0,\;\;\lambda_{\text{HADEP}}^{\text{T}1999}=-0.071,\\ \lambda_{\text{ANAP}}^{\text{T}1999}=0.34,\;\;\lambda_{\text{CHP}}^{\text{T}1999}\equiv 0.73,\\ \lambda_{\text{DSP}}^{\text{T}1999}=0.72,\;\;\beta_{1999}^{\text{T}}=0.38. \end{array}$$

The *β*-coefficient and the valence estimates of DSP and MHP and CHP are significantly nonzero. The probability that a Turkish voter chooses FP with lowest valence in 1999, when all parties locate at the mean, *ρ*_FP_^T1999^ in (14), is

(50) ρFPT1999=[∑k=17exp⁡[λjT1999−λFPT1999]]−1=[1+e0.82+e0.16+e0.09+ e0.5+e0.89+e0.88]−1≃0.08.

Given that 2*β*_1999_^T^(1 − 2*ρ*_FP_^T1999^) = 2 × 0.38 × 0.84 = 0.64 and since *σ*_T1999_^2^ = 2.34 in (48), then using (15), Turkey's convergence coefficient in 1999, in Table 4, is

(51) c1999T=2β1999T(1−2ρFPT1999)σT19992=0.64×2.34=1.49.

The convergence coefficient is significantly higher that 1 and significantly lower than 2 (see Appendix A.2). From (17) FP's Hessian at the origin is

(52) CFPT1999=2β1999T(1−2ρFPT1999)∇!999T−I=0.64[1.200.780.781.14]−I=[−0.240.4480.448−0.27].

When at the electoral origin, FP's characteristic function shows that its vote share function is at a saddlepoint as the eigenvalues of *C*_FP_^T1999^ are −0.74 with minor eigenvector (+1 − 1.116) and +0.23 with major eigenvector (+1, +0.896). Moreover, as seen in Appendix A.2, the 95% confidence bounds show that at the lower bound of *C*_FP_^T1999^ FP has no incentive to move but it does at the upper bound. Since FP wants to move at the central estimate of *C*_FP_^T1999^ in (52) it is probable that in general FP wants to move away from the mean to increase its vote share. Moreover, since the convergence coefficient is significantly greater than 2, then with a high degree confidence, the electoral mean *cannot* be a LNE for Turkey in 1999.

The electoral covariance matrix of the *2002 Turkish election* is

(53) $$\begin{array}{c} \nabla_{2002}^{\text{T}}=\left[\begin{array}{cc} \sigma_{R}^{2}=1.18 & \sigma_{RN}=0.74\\ \sigma_{NR}=0.74 & \sigma_{N}^{2}=1.15 \end{array}\right], \end{array}$$

with *σ*_T2002_^2^ = trace (∇_2002_^T^) = 2.33.

Note that the covariance matrix of 1999 in (48) and that of 2002 in (53) suggest few changes in the distribution of voters between these two election. Figures 8 and 9 suggest that there were few changes in party positions between these two elections. The basis of support for the AKP may be regarded as similar to that of the banned FP, suggesting that the leader of this party changed the party's position on the religion axis, adopting a much less radical position. One would think of this as generating political stability in Turkey. Yet, between 1999 and 2002, Turkey experienced two severe economic crises and in 2002, a 10% electoral cut-off rule was instituted. The crises and the cut-off rule changed the political landscape in Turkey. In the 2002 election, seven parties obtained less than 10% of the vote and won no seats. The AKP won 34% of the vote, and due to the cut-off rule, obtained a majority of the seats (363 out of 550).

Our analysis reflects this change in the political landscape. Using DYP as the base party, from Table 3, the 2002 MNL coefficients are

(54) $$\begin{array}{c} \lambda_{\text{ANAP}}^{\text{T}2002}=-0.31,\;\;\lambda_{\text{MHP}}^{\text{T}2002}=-0.12,\\ \lambda_{\text{DYP}}^{\text{T}2002}\equiv 0.0,\;\;\lambda_{\text{HADEP}}^{\text{T}2002}=0.43,\\ \lambda_{\text{AKP}}^{\text{T}2002}=0.78,\;\;\lambda_{\text{CHP}}^{\text{T}2002}\equiv 1.33,\;\;\beta_{2002}^{\text{T}}=1.52. \end{array}$$

The *β*-coefficient and the valences of AKP and CHP are significantly nonzero with ANAP having the lowest valence. The probability of voting ANAP, when parties locate at the mean, *ρ*_ANAP_^T20029^ in (14), is

(55) ρANAPT2002=[∑k=16exp⁡[λjT2002−λANAPT2002]]−1=[1+e0.19+e0.31+e0.74+ e1.09+e1.164]−1≃0.08.

Given that 2*β*_2002_^T^(1 − 2*ρ*_ANAP_^T2002^) = 2 × 1.52 × 0.84 = 2.55 and since *σ*_T2002_^2^ = 2.33 from (53), then using (15) we find that the 2002 convergence coefficient for Turkey, in Table 4, is

(56) $$\begin{array}{c} c_{2002}^{\text{T}}=2\beta_{2002}^{\text{T}}\left(1-2\rho_{\text{ANAP}}^{\text{T}20029}\right)\sigma_{\text{T}2002}^{2}=2.55\times 2.33=5.94. \end{array}$$

The political changes induced by the cut-off rule led to a higher convergence coefficient in 2002 relative to 1999 (increasing from a low of *c*_1999_^T^ = 1.49 in (51) to a high *c*_2002_^T^ = 5.94 in (56)). An indication that a more fractionalized polity emerged from this reform. The convergence coefficient of the 2002 election is significantly above 2, the dimension of the policy space (see Appendix A.2) giving ANAP an incentive to locate far from the mean. ANAP's characteristic matrix using (17) is

(57) CANAPT2002=2β2002T(1−2ρANAPT2002)∇2002T−I=2.55[1.180.740.741.15]−I=[2.011.881.881.93].

When at the origin, *C*_ANAP_^T2002^ indicates that ANAP is minimizing its vote share since its eigenvalues are both positive (0.090 and 3.850). This together with the 95% confidence bounds in Appendix A.2 implies that there is a high probability that ANAP will vacate the center and that the mean is *not* an LNE for Turkey in 2002.

#### 3.2.3. The 1997 Polish Election

In the election held in Poland in 1997 (In this election Poland used an open-list proportional representation electoral system with a threshold of 5% nationwide vote for parties and 8% for electoral coalitions. Votes are translated into seats using the D'Hondt method.) the following five parties won seats in the Sejm (lower house). The left-wing excommunist Democratic Left Alliance (SLD) and the agrarian Polish Peoples' Party (PSL), both of which have been the most frequent governing parties in the postcommunist period. The Freedom Union (UW) and the Solidarity Election Action (AWS) had grown out of the Solidarity movement. AWS combined various mostly right wing and Christian groups under one label, while UW was formed based on the liberal wing of Solidarity. The remaining party is the Movement for Reconstruction of Poland (ROP).

Applying factor analysis to questions from the Polish National Election Survey an economic and a social value dimensions were identified (see [40]). The *economic* dimension is influenced by issues such as privatization versus state ownership of enterprises, fighting unemployment versus keeping inflation and government expenditure under control, proportional versus flat income tax, support versus opposition to state subsidies to agriculture, and state versus individual social responsibility. The separation of church and state versus the influence of church over politics, complete decommunization versus equal rights for former nomenclature, and abortion rights regardless of situation versus no such rights regardless of situation are the most influential issues in this *social values* dimension. The distribution of voters along these dimensions is seen in Figure 9. (See Schofield et al. [40] for details of the estimation.)

The covariance matrix for the *1997 Polish* (P) *election* is

(58) $$\begin{array}{c} \nabla_{1997}^{\text{P}}=\left[\begin{array}{cc} \sigma_{E}^{2}=1.00 & \sigma_{ES}=0.0\\ \sigma_{SE}=0.0 & \sigma_{S}^{2}=1.00 \end{array}\right], \end{array}$$

with variance *σ*_P1997_^2^ = trace(∇_1997_^P^) = 2.00.

From Table 3, the MNL coefficients for the 1997 election are

(59) $$\begin{array}{c} \lambda_{\text{UPR}}^{\text{P}1997}=-2.3,\;\;\lambda_{\text{UP}}^{\text{P}1997}=-0.56,\\ \lambda_{\text{ROP}}^{\text{P}1997}\equiv 0.0,\;\;\lambda_{\text{PSL}}^{\text{P}1997}=0.07,\\ \lambda_{\text{UW}}^{\text{P}1997}\equiv 0.73,\;\;\lambda_{\text{SLD}}^{\text{P}1997}=1.40,\\ \lambda_{\text{AWS}}^{\text{P}1997}=1.92,\;\;\beta_{1997}^{\text{P}}=1.74. \end{array}$$

The *β*-coefficient and valence estimates for all parties except UP and PSL are significantly nonzero. The probability of voting UPR with lowest valence, in 1997, when parties locate at the mean, *ρ*_TW_^P1997^ in (14), is

(60) ρUPRP1997=[∑k=16exp⁡[λjP1997−λUPRP1997]]−1=[1+e0.048+e3.08+e4.27+ e3.77+e2.42]−1≃0.01.

Given that 2*β*_1997_^P^(1 − 2*ρ*_UPR_^P1997^) = 2 × 1.74 × 0.98 = 3.41 and since *σ*_P1997_^2^ = 2 from (58), then using (15) the convergence coefficient for Poland, in Table 4, is

(61) c1997P=2β1997P(1−2ρUPRP1997)σP19972=3.41×2=6.82.

Appendix A.2 shows that *c*_1997_^P^ = 6.82 is significantly greater than 2 and thus fails the necessary condition for convergence to the mean. UPR's Hessian from (17) is

(62) CUPRP1997=2β1997P(1−2ρUPRP1997)∇1997P−I=3.41[1.00.00.01.0]−I=[2.410.00.02.41].

The trace (= 3.82), the determinant (= 5.80), and the eigenvalues of *C*_UPR_^I^  (2.41,1.41) are positive. The 95% confidence bound of *C*_UPR_^I^ in Appendix A.2 also shows positive eigenvalues at the lower and upper bounds of *C*_UPR_^I^. Thus, with a high degree of certainty UPR locates far from the origin to maximize its votes and the electoral mean is *not* a LNE for 1997 Polish election.

Summarizing, in this section we examined three countries that use proportional representation. Their convergence coefficients are significantly higher than 2, the dimension of the policy space and are also much higher than that of the US and the UK. A high convergence coefficient signals then a high degree of political fractionalization in these multi-party parliamentary democracies.

### 3.3. Convergence in Anocracies

We now study elections in Georgia, Russia, and Azerbaijan. In these partial democracies or anocracies, (The term “partial democracy” has been applied to new democracies lacking the full array of democratic institutions present in western democracies (see [41].)) the President/autocrat holds regular presidential and legislative elections while exerting undue influence on the elections. Anocracies lack important democratic institutions such as freedom of the press. Autocrats hold regular elections in an attempt to give their regime legitimacy. The autocrat “buys” legitimacy by rewarding their supporters and opposition members with well-paid legislative positions and give legislators the ability to influence policies. Opposition parties participate in elections to become known political entities. This allows them to regularly communicate with voters. Their objective is to oust the autocrat either in a future election or through popular uprisings. We assume that opposition parties maximize their vote share even when understanding that there is little chance of ousting the autocrat in the election.

#### 3.3.1. The 2008 Georgian Election

**Figure 10:**
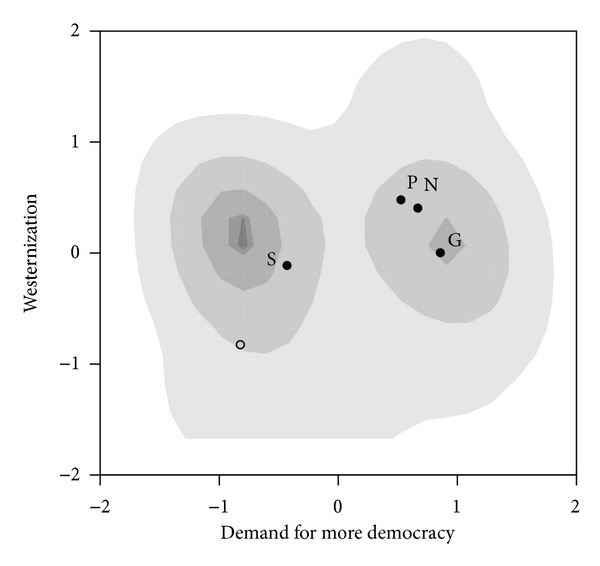
Voter distribution and candidate positions in the 2008 Georgian election.

We use the postelection survey conducted by GORBI-GALLUP International from March 19 through April 3, 2008, to built a formal model of the 2008 election in Georgia (see [42]). The factor analysis done on the survey questions determined that there were two dimensions describing voters' attitudes towards democracy and the west. One dimension is strongly related with the respondents' attitude toward the US, the EU and NATO with larger values in the West (*W* = *y*-axis) dimension implying a stronger anti-western attitude. Along the democracy (*D* = *x*-axis) dimension larger values are associated with negative judgements on the current state of democratic institutions in Georgia, coupled with a demand for more democracy. The electoral distribution along these two dimensions is given in Figure 10. The points (S, G, P, N) in Figure 10 represent the estimated positions of the four candidates: Saakashvili (S), Gachechiladze (G), Patarkatsishvili (P), and Natelashvili (N). (See Schofield et al. [39] for details of the estimation.)

The 2008 electoral covariance matrix in the Democracy (*D*) and West (*W*) axes is

(63) $$\begin{array}{c} \nabla_{2008}^{\text{G}}=\left[\begin{array}{cc} \sigma_{D}^{2}=0.82 & \sigma_{DW}=0.03\\ \sigma_{WD}=0.03 & \sigma_{W}^{2}=0.91 \end{array}\right] \end{array}$$

with *σ*_G2008_^2^ ≡ trace (∇_2008_^G^) = 1.73.

From Table 5, the MNL estimates of the 2008 election with Natelashvili as the base candidate are

(64) $$\begin{array}{c} \lambda_{\text{S}}^{\text{G}2008}=2.56,\;\;\lambda_{\text{G}}^{\text{G}2008}=1.50,\;\;\lambda_{\text{P}}^{\text{G}2008}=0.53,\\ \lambda_{\text{N}}^{\text{G}2008}\equiv 0.0,\;\;\beta_{2008}^{\text{G}}=0.78. \end{array}$$

All coefficients are significantly nonzero showing Natelashvili as having the lowest valence.

The probability that a Georgian votes for Natelashvili, when all candidates locate at the mean, is

(65) ρNG2008=[∑k=14exp⁡[λjG2008−λNG2008]]−1=[1+e2.56+e1.50+e0.53]−1≃0.05.

Given that 2*β*_2008_^G^(1 − 2*ρ*_N_^G2008^) = 2 × 0.78 × 0.9 = 1.4 and since *σ*_G2008_^2^ = 1.73 from (63), then using (15) Georgia's the convergence coefficient, in Table 6, is

(66) c2008G=2βG2008(1−2ρNG2008)σG20082=1.4×1.73=2.42.

As shown in Appendix A.3, *c*_2008_^G^ is *not* significantly different from 2 and thus fails the necessary condition for convergence to the mean. Natelashvili's Hessian or characteristic matrix, from (17), is

(67) CNG2008=2β2008G(1−2ρNG2008)∇2008G−I=1.4[0.820.030.030.91]−I=[0.150.040.040.28].

Since the eigenvalues of *C*_N_^G2008^ are both positive (+0.139, +0.291), Natelashvili's vote share function is at a minimum when he is at the mean and has an incentive to move to increase his vote share. This together with the analysis of the 95% confidence intervals of *C*_N_^G2008^ in Appendix A.3 shows that with a high degree of certainty Natelashvili will locate far from the mean. This is not surprising since Georgians managed to induce three major changes in government through mass protests prior to this election. Thus, with a high degree of certainty Natelashvili locates far from the origin in this election and the electoral mean *cannot* be an LNE for the 2008 Georgian election.

#### 3.3.2. The 2007 Russian Election

**Figure 11:**
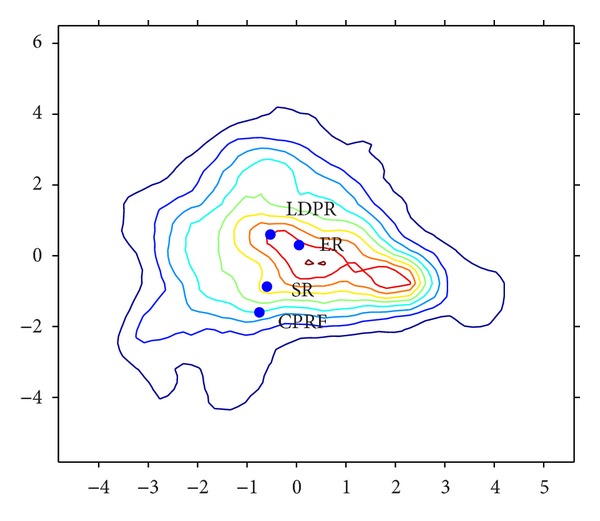
Party positions and voters distribution in the 2007 Russian election.

The analysis of the 2007 Russian election concentrates on four parties: the pro-Kremlin United Russia party (ER), Liberal Democratic Party (LDPR), Communist Party (CPRF), and Fair Russia (SR). Voters' ideological preferences were measured according to two questions taken from the survey conducted by VCIOM (Russian Public Opinion Research Center) in May 2007 (see [43]). The first dimension gives a measure of voters general (dis)satisfaction (*D* = *x*-axis). High values in this dimension correspond to negative feelings toward “justice,” “labor” and, to a lesser extent, “order,” “state,” “stability,” and “equality.” Also, those with high values of the first axis tend to feel neutral toward order, elite, West, and non-Russians. The second dimension measures the voter's degree of economic liberalism (*E* = *y*-axis). High values correspond to positive feelings to “freedom,” “business,” “capitalism,” “well-being,” “success,” and “progress,” and to negative feelings toward “communism,” “socialism,” “USSR,” and related concepts. The distribution of voter preferences along these two dimensions can be seen in Figure 11. (See Schofield and Zakharov [43] for details of the estimation.)

The 2007 electoral covariance matrix along the (dis) satisfaction (*D*) and economic liberalism (*E*) axes is

(68) $$\begin{array}{c} \nabla_{2007}^{\text{R}}=\left[\begin{array}{cc} \sigma_{D}^{2}=2.95 & \sigma_{DE}=0.13\\ \sigma_{ED}=0.13 & \sigma_{E}^{2}=2.95 \end{array}\right], \end{array}$$

with *σ*_R2007_^2^ ≡ trace(∇_2007_^R^) = 5.9.

From Table 5, the MNL estimates of the spatial model for Russia are

(69) $$\begin{array}{c} \lambda_{\text{SR}}^{\text{R}2007}=-0.4,\;\;\lambda_{ER}^{\text{R}2007}\equiv 0,\;\;\lambda_{\text{LDPR}}^{\text{R}2007}=0.153,\\ \lambda_{\text{CPRF}}^{\text{R}2007}=1.971,\;\;\beta_{2007}^{\text{R}}=0.181. \end{array}$$

Distance and all valences, except for that of the LDPR party, are significantly nonzero. When parties locate at the mean, the probability that a Russian votes for Fair Russia (SR) with lowest valence, from (14) is

(70) ρSRR2007=[∑k=14exp⁡[λjR2007−λSRR2007]]−1=[1+e0.4+e0.553+e2.371]−1≃0.07.

Given that 2*β*_2007_^R^(1 − 2*ρ*_SR_^R2007^) = 2 × 0.181 × 0.86 = 0.31 and since *σ*_R2007_^2^ = 5.9 from (68), then using (15) Russia's convergence coefficient, in Table 6, is

(71) c2007R=2β2007R(1−2ρSRR2007)σR20072=0.31×5.9=1.83.

Since *c*_2007_^R^ is not significantly different from 2 (see Appendix A.3), the necessary condition for convergence is *not* met. The characteristic matrix or Hessian of Fair Russia (SR) from (17) is

(72) CSRR2007=2β2007R(1−2ρSRR2007)∇2007R−I=0.31[2.950.130.132.95]−I=[−0.0860.040.04−0.086].

The eigenvalues are both negative (−0.126, −0.046), implying that at this central estimate Fair Russia is maximizing its vote share and thus has no incentive to vacate the origin. This conclusion holds at the lower 95% bound of *C*_SR_^R2007^ in Appendix A.3. However, at the upper bound of *C*_SR_^R2007^ Fair Russia is minimizing its vote share. It seems then that with the Russian President and his party exerting much influence over the election and Putin being so popular that Fair Russia is more likely to remain at the origin. (This result however highlights that unexpected political events could prompt Fair Russia to move from the origin.) It is then likely that the electoral mean is a LNE for the 2007 Russian election.

#### 3.3.3. The 2010 Election in Azerbaijan

In the 2010 election in Azerbaijan, 2,500 candidates filed application to run in the election, but only 690 were given permission by the electoral commission. The parties that competed in the election were the Yeni Azerbaijan Party (the party of the President, YAP), Civic Solidarity Party (VHP), Motherland Party (AVP), Azerbaijan Popular Front Party (AXCP), and Musavat (MP). Various small parties formed political blocks.

President Ilham Aliyev's ruling Yeni Azerbaijan Party took a majority of 72 out of 125 seats. Nominally independent candidates, who were aligned with the government, received 38 seats, and 10 small opposition or quasiopposition parties took 10 seats. The Democratic Reforms party, Great Creation, the Movement for National Rebirth, Umid, Civic Welfare, Adalet (Justice), and the Popular Front of United Azerbaijan most of which were represented in the previous parliament, won one seat a piece. Civic Solidarity retained its 3 seats and Ana Vaten kept the 2 seats they had in the previous legislature. For the first time, not a single candidate from the opposition Azerbaijan Popular Front (AXCP) or Musavat were elected.

We organized a small preelection survey of 2010 election in Azerbaijan allowing us to construct a model of the election (see [42]). For VHP and AVP, the estimation of their party positions was very sensitive to inclusion or exclusion of one respondent. Thus, we used only the small subset of 149 voters who completed the factor analysis questions and intended to vote for YAP or the AXCP+MP coalition.

**Figure 12:**
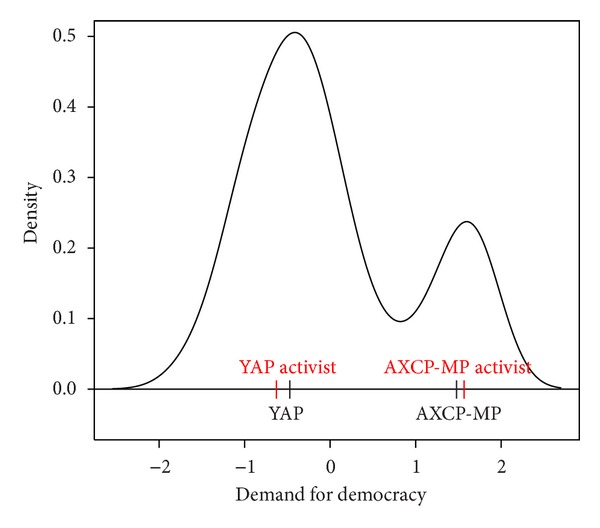
Voter distribution and activist positions in the 2010 Azerbaijani election.

The factor analysis showed that voters were only concerned with *one dimension*: the “demand for democracy” with higher values being associated with voters who had a negative evaluation of the current democratic situation in Azerbaijan, who did not think that free opinion is allowed, had a low degree of trust in key national political institutions, and expected that the 2010 parliamentary election would be undemocratic.  Figure 12 shows the distribution of voters and the party positions at the mean of their supporters. (See [42] for details of the estimation.) In this one dimensional model the variance is

(73) $$\begin{array}{c} \sigma_{\text{A}2010}^{2}\equiv \text{trace}\left(\nabla_{\text{G}}^{2010}\right)=0.93. \end{array}$$

The *binomial* logit estimates for the 2010 election with AXCP-MP as the base party, in Table 5, are

(74) $$\begin{array}{c} \lambda_{\text{YAP}}^{\text{A}2010}=1.30,\;\;\lambda_{\text{AXCP-MP}}^{\text{A}2010}\equiv 0.0,\;\;\beta_{2010}^{\text{A}}=1.34. \end{array}$$

All coefficients are significantly nonzero with AXCP-MP having the lowest valence. If these two parties locate at the mean, the probability that an Azerbaijani votes AXCP-MP from (14) is

(75) ρAXCP-MPA2010=[∑k=12exp⁡[λjA2010−λAXCP-MPA2010]]−1=[1+e1.3]−1≃0.21.

Given that 2*β*_2010_^A^(1 − 2*ρ*_AXCP-MP_^A2010^) = 2 × 1.34 × 0.58 = 1.554 and since *σ*_A2010_^2^ = 0.93 from (73), then using (15) the convergence coefficient for Azerbaijan, in Table 6, is

(76) c2010A=2β2010A(1−2ρAXCP-MPA2010)σA20102=1.554×0.93=1.445.

Given that *c*_2010_^A^ is not significantly different from 1, the dimension of the policy space (see Appendix A.3) and the necessary condition for convergence is *not* met. The one dimensional Hessian of AXCP-MP from (17) is

(77) CAXCP-MPA2010=2β2010A(1−2ρAXCP-MPA2010)σA20102−I=1.554×0.93−1=0.445.

Clearly, *C*_AXCP-MP_^A2010^ has a single positive eigenvalue indicating the AXCP+MP is minimizing its vote share at the origin. The 95% bounds of *C*_AXCP-MP_^A2010^ in Appendix A.3 shows that this matrix has positive eigenvalues at the lower and upper bounds of the confidence interval. Thus, with a high degree of certainty AXCP+MP will deviate from the origin and the electoral mean is *not* a LNE for the 2010 election in Azerbaijan.

This section illustrates that for the three anocracies that we consider the convergence coefficient does not satisfy the necessary condition for convergence to the mean. That is, these convergence coefficients are not significantly different from the dimension of the policy space. As a consequence, parties are at a knife-edge equilibrium. Under some conditions, parties converge to the mean, under others they diverge. Which equilibrium materializes depends on how popular or unpopular the President/autocrat and his party are and so depends on the valence of all parties and on how dispersed voters are in the policy space. Thus any change in valence can substantially affect party positions.

## 4. Convergence across Political Systems

In the previous sections we used the unifying framework of Schofield's [9] stochastic electoral model outlined in Section 2 to study whether parties locate near or far from the electoral mean for countries with plurality and proportional representation systems and in anocracies. Using this framework we estimated the convergence coefficient for various elections in different countries. We will now use this dimensionless coefficient to compare convergence to the electoral mean across elections, countries, and political systems. We can then illustrate the use of the convergence coefficient to classify political systems.  Table 7 presents a summary of the convergence coefficients across elections, countries, and political systems that we now discuss.

As Table 7 indicates the two countries using plurality systems (the US and the UK) studied in Section 3.1 meet the conditions for convergence to the mean. Thus, suggesting that plurality rule imposes a strong centripetal tendency that keeps parties close to the mean. Our analysis suggests that in countries with plurality systems the convergence coefficient will be low at or below the dimension of the policy space.

Of the anocratic countries that we studied in Section 3.3, Georgia seems to have the highest convergence coefficient, *c*_2008_^G^ = 2.42 in (66) which is not different from 2, suggesting that parties can diverge from the mean. (Note that prior to 2008 Georgians had already brought about three major political changes through mass popular revolt. This rebellious “tradition” may give opposition candidates the ability to position themselves away from the mean.) The convergence coefficient of all three anocracies was not significantly different than the dimension of the policy space [2 for Georgia and Russia and 1 for Azerbaijan: *c*_2008_^G^ = 2.42 given in (66), *c*_2007_^Ru^ = 1.83 in (71), and *c*_2010_^A^ = 1.44 in (76)]. These results suggest that convergence in anocracies is fragile and depends on the distribution of voters' preferences as well as on the valences of the autocrat and the opposition parties.

The countries with proportional systems studied in Section 3.2 have convergence coefficients that are significantly above their two-dimensional policy space signalling the lack of convergence of small valence parties to the electoral mean (from Table 7, Israel's *c*_1996_^I^ = 4.06 in (46), Turkey's *c*_1999_^T^ = 1.49 in (51) in 1999, and *c*_2002_^T^ = 5.94 in (56) in 2002 and Poland's *c*_1997_^P^ = 6.82 in (61)). Having no possibility of forming government, these small parties maximize their vote shares by locating closer to their core supporters. Elections lead to multiparty legislatures producing a highly fragmented party system where coalition governments are the norm. Note that changes to the electoral process in Turkey between 1999 and 2002 forced parties to move from locating close to the mean in 1999 to diverging towards their partisan constituencies so as to increase their vote shares in 2002. These results suggest that in countries with proportional systems, with highly fragmented political parties, divergence from the mean is the norm.

We can explain the lack of convergence to the mean in proportional systems with multiparty (>3) legislatures by noting that the convergence coefficient *c* ≡ *c*(***λ***, *β*, *σ* ^2^) = 2*β*[1 − 2*ρ*_1_]*σ* ^2^ in (15) depends on fundamental characteristics of the electorate. These characteristics include the weight given by voters to the distance to the parties' positions, *β*; the electoral variance, *σ* ^2^ in (16); and the probability that a voter chooses the lowest valence party, *ρ*_1_ in (14). Thus, in countries with many parties, the smallest low valence parties have little chance of receiving much support, a low *ρ*_1_. If, in addition, voters care a lot about policy differences (a high *β*) and if the electorate is very dispersed (a high *σ* ^2^), then small parties will have an incentive to move towards their core supporters and away from the mean. That is, in highly fragmented polities where voters and correspondingly parties are very dispersed, we observe high convergence coefficients.

In essence, Schofield's [9] Valence theorem gives a simple summary statistic, the convergence coefficient, that measures the degree of fragmentation, or lack thereof, in each polity. Poland is an extreme case of this fragmentation and correspondingly has a very high convergence coefficient (see Table 7).

The are other measures of political fragmentation in the literature. The *effective number of party vote strength* (*env*) used by Laakso and Taagepera [15] serves to measure how many dominant parties there are in a polity a given election. To find the *env*, let the Herfindahl index of the election be given by

(78) $$\begin{array}{c} H_{v}=\sum_{j=1}^{p}v_{j}^{2}, \end{array}$$

where *v*_*j*_ is the vote share of party *j* for *j* = 1,…, *p*. This Herfindahl index *H*_*v*_ gives a measure of the party size in an election and measures how competitive the election was. Laakso and Taagepera's *effective number of party vote strength* is then the inverse of *H*_*v*_; that is,

(79) $$\begin{array}{c} env=H_{v}^{-1}. \end{array}$$

In the same way we can define the *effective number of party seat strength* (*ens*) using seat shares instead of vote shares giving us a measure of the strength of parties in a legislature.

We calculate the *env* and *ens* for each election we consider (see Table 7) using all the parties that obtained votes in each election and exclude parties that ran in the election but that got no votes. We now compare the level of fragmentation given by the *env* and *ens* with that given by the convergence coefficient for each country and each election under the three political systems that we studied.

We first examine countries with plurality rule. In Table 7 we see that for the US, the *env* and the *ens* at the Presidential and House levels are closely aligned. There is little variation between the *env* and *env* indices in the three elections. According to these indices there is essentially no change in political fragmentation across these three elections. The convergence coefficient however rises in 2008 relative to 2000 and 2004 indicating that in 2008 the dispersion among voters was higher than in the previous two elections. For the US, the convergence coefficient provides more information than do *env* or *env*. For the UK, the convergence coefficient shows that the electorate was more dispersed in 2010 than in 2005 (see Tables 2 and 7). This dispersion led to the first minority government since 1974 which resulted in higher effective number of parties as measured by the *env* and *env*. All three measures, *c*, *env*, and *ens*, indicate that the United Kingdom became more fragmented in 2010. Thus, in the countries using plurality, the convergence coefficient tends to provide more information than the *env* and *ens* numbers do as the convergence coefficient takes into account the degree of dispersion among the electorate and the valence of parties.

Polities with high convergence coefficients (Israel, Turkey in 2002 and Poland in Table 7) had a large number of parties competing in these elections. The greater the number of parties obtaining votes, and thus effectively competing in the election, led to large *env* values. These elections produced highly fragmented legislatures leading to very high *ens* values. Having a large number of effective parties competing in the election and greater effective number of parties in the legislature does not necessarily translate into a higher convergence coefficient. The convergence coefficient is lower for Israel with a larger number of effective parties (higher *env* and *ens*) than for Poland with fewer parties. Changes in the Turkish electoral system between 1999 and 2002 in which a minimum cut-off rule has instituted led to a high *env* but a low *ens*. Small parties were however able to gain enough votes leading to a high convergence coefficient, an indication that these parties would disperse themselves in the policy space. The *env* and *ens* values of the 2002 Turkish election show high party fragmentation but no legislative fragmentation. This shows that these three measures of fragmentation provide different information about a particular election.

The convergence coefficient suggests that a way of interpreting the arguments of Duverger [44] and Riker [45] on the effects of proportional electoral methods on electoral outcomes: the strong *centrifugal* tendency pulling all parties away from the electoral mean towards their core constituency. This tendency will be particularly strong for small, or low valence, parties. In particular, even small parties in such a polity can assign a nonnegligible probability to becoming a member of a coalition government, and it is this phenomenon that maintains the fragmentation of the party system. For example, in Poland no party can obtain a majority and parties and coalitions regularly form and dissolve. In general the convergence coefficients in Poland were of the order of 6.0 in the elections in the 1990's.

For countries using proportional representation, while the *env* and *ens* give a measure of electoral and legislative dispersion, the convergence coefficient provides a measure that summarizes dispersion across voters and parties in the policy space.

In the anocratic countries studied, the convergence coefficient seems in line with the *env* in presidential elections but going in the opposite direction in parliamentary elections (see Table 7). In these countries, the convergence coefficient does not meet the necessary condition for convergence to the mean. These countries that we study show that parties could either converge to or diverge from the mean under anocracy as the equilibrium is fragile. Changes in valences, for example, of the autocrat or in voters' preferences, can lead small valence opposition parties to diverge from the mean and to mount popular uprisings as happened in previous elections in Georgia or in recent Arab uprisings.

The convergence coefficient reflects information that the *env* and *ens* cannot capture as it reflects the preferences of the electorate through the policy weight, *β*; the perceived ability of parties or candidates to govern as captured by their valences ***λ*** = (*λ*_1_,…, *λ*_*p*_); and the dispersion of voters' preferences in the policy space, *σ* ^2^. All of which are not taken into account in the *env* and *ens*. Moreover, *env* and *ens* have nothing to say about the dispersion in parties' positions relative to the mean.

The analysis carried out in this section suggests that there is an inverse relationship between the degree of fractionalization in a polity and the convergence coefficient. By our interpretation of the nature of the convergence coefficient, the convergence effect in presidential elections in the United States is stronger than in parliamentary elections in Great Britain. That is, our results suggest that democratic presidential systems have fewer parties and a low convergence coefficient. Parliamentary democracies operating under plurality rule tend to have more parties than presidential democracies and a somewhat higher convergence coefficient. Parliamentary democracies operating under proportional representation tend to have multiparty legislatures and high convergence coefficients. Anocratic countries tend to have multiple parties competing in the election but low convergence coefficients as opposition parties remain close to the electoral mean when Presidents/autocrats have high valences and diverge when they do not.

## 5. Conclusion

In this paper, Schofield's [9] Valence Theorem together with multinomial logit models of elections are used as a unifying framework to compare the convergence properties of parties across elections, countries, and political systems. We found evidence to support the hypothesis that in countries with proportional representation parties located away from the electoral mean.

We relate the convergence coefficient to the effective number of parties according to both vote (*env*) and seat (*ens*) shares and showed how the characteristics of the electorate and the political regime under which parties operate. Then, compare the convergence coefficient to the fractionalization measures provided by the *env* and *ens*. The advantage of the convergence coefficient is that it is a summary statistic that incorporates the preferences of voters, the valence of parties, and the dispersion of voters and parties in the policy space.

## Figures and Tables

**Table 1:** MNL spatial model for countries with plurality systems.

	United States^b^				United Kingdom^c^		
	Party	2000	2004	2008	Party	2005	2010
Var		Est.^a^ |*t* − value|	Est.^a^ |*t* − value|	Est.^a^ |*t* − value|		Est.^a^ |*t* − value|	Est.^a^ |*t* − value|
*β*		0.82*** (14.9)	0.95*** (14.21)	0.85*** (14.16)		0.15*** (12.56)	0.86*** (38.45)

Valence	*λ*_rep_	−0.43*** (5.05)	−0.43*** (5.05)	−0.84*** (7.64)	*λ*_Lab_	0.52*** (6.84)	−0.04 (1.31)
					*λ*_Con_	0.27*** (3.22)	0.17*** (4.50)

Base party		Dem^b^	Dem^b^	Rep^b^		Lib^c^	Lib^c^
*n*		1,238	935	788		1149	6218
*LL*		−708	−501	−298		−1136	−5490

^a∗^prob < 0.05; **prob < 0.01; ***prob < 0.001.

^b^US: Rep: Republican; Dem: Democrats.

^c^UK: Lab: Labour; Con: Conservatives; Lib: Liberal Democrats.

**Table 2:** The convergence coefficient in plurality systems.

	United States			United Kingdom	
	2000	2004	2008	2005	2010
Weight of policy differences (*β*)					
Est. *β* (conf. Int.^a^)	0.82 (0.71, 0.93)	0.95 (0.82, 1.08)	0.85 (0.73, 0.97)	0.15 (0.13, 0.17)	0.86 (0.81, 0.90)

Electoral variance (trace∇ = *σ* ^2^)					
*σ* ^2^	1.17	1.17	1.63	5.607	1.462

Probability of voting for lowest valence party (party 1, *ρ*_1_ = [∑_*k*=1_^*p*^‍exp⁡(*λ*_*k*_−*λ*_1_)]^−1^)					
	Dem^b^	Dem^b^	Rep^b^	LibDem^c^	Labour^c^
Est. *ρ*_1_ (conf. Int.^a^)	*ρ*_Dem_ = 0.4 (0.35, 0.44)	*ρ*_Dem_ = 0.4 (0.35, 0.44)	*ρ*_rep_ = 0.3 (0.26, 0.35)	*ρ*_Lib_ = 0.25 (0.18, 0.32)	*ρ*_Lab_ = 0.32 (0.29, 0.32)

Convergence coefficient (*c* ≡ *c*(***λ***, *β*, *σ* ^2^) = 2*β*[1 − 2*ρ*_1_]*σ* ^2^)					
Est. *c* (conf. Int.^a^)	0.38 (0.2, 0.65)	0.45 (0.23, 0.76)	1.1 (0.71, 1.52)	0.84 (0.51, 1.25)	0.98 (0.86, 1.10)

^a^Conf. Int.: confidence intervals.

^b^US: Dem: Democrats; Rep: Republican.

^c^UK: LibDem: Liberal Democrats.

**Table 3:** MNL spatial model for countries with proportional systems.

Var	Israel^b^		Turkey^d^			Poland^c^	
	Party	1996	Party	1999	2002	Party	1997
Distance		Est.^a^ |*t* − value|		Est.^a^ |*t* − value|	Est.^a^ |*t* − value|		Est.^a^ |*t* − value|
*β*		1.207*** (18.43)		0.375*** (4.26)	1.52*** (12.66)		1.739*** (15.04)

Valence	*λ*_Lik_	0.777*** (4.12)	*λ*_DSP_	0.724*** (4.73)		*λ*_SLD_	1.419*** (7.47)
	*λ*_Lab_	0.999**** (6.06)	*λ*_MHP_	0.666*** (4.53)	−0.12 (0.66)	*λ*_PSL_	0.073 (0.33)
	*λ*_NRP_	−0.626*** (2.53)	*λ*_FP_	−0.159 (0.90)		*λ*_AWS_	1.921*** (11.05)
	*λ*_MO_	−1.259*** (4.38)	*λ*_ANAP_	0.336*** (2.19)	−0.31 (1.63)	*λ*_UW_	0.731*** (3.67)
	*λ*_TW_	−2.291*** (8.30)	*λ*_CHP_	0.734*** (4.12)	1.33*** (7.40)	*λ*_UP_	−0.56*** (2.13)
	*λ*_Shas_	−2.023*** (6.45)	*λ*_HADEP_	−0.071 (0.30)	0.43* (2.0)	*λ*_UPR_	−2.348*** (4.69)
			*λ*_AKP_		0.78*** (5.2)		

Base party		Meretz		DYP^d^	DYP^d^		ROP^c^
*n*		922		635	483		660
*LL*		−777		−1183	−737		−855

^a∗^prob < 0.05; **prob < 0.01; ***prob < 0.001.

^b^Israel: Lik: Likud; Lab: Labor; NRP: Mafdal; Mo: Moledet; TW: Third Way.

^c^Poland: SLD: Democratic Left Alliance; PSL: Polish People's Party; UW: Freedom Union; AWS: Solidarity Election.

Action; UP: Labor Party; UPR: Union of Political Realism; ROP: Movement for Reconstruction of Poland; SO: Self Defense; PiS: Law and Justice; PO: Civic Platform; LPR: League of Polish Families; DEM: Democratic Party; SDP: Social Democracy of Poland.

^d^Turkey: DSP: Democratic Left Party; MHP: Nationalist Action Party; FP: Virtue Party; ANAP: Motherland Party; CHP: Republican People's Party; HADEP: People's Democracy Party; DYP: True Path Party.

**Table 4:** The convergence coefficient in proportional systems.

	Israel	Turkey		Poland
	1996	1999	2002	1997
Weight of policy differences (*β*)				
Central Est.^a^ of *β* (conf. Int.^b^)	1.207 (1.076, 1.338)	0.375 (0.203, 0.547)	1.520 (1.285, 1.755)	1.739 (1.512, 1.966)

Electoral variance (trace∇ = *σ* ^2^)				
*σ* ^2^	1.732	2.34	2.33	2.00

Probability of voting for lowest valence party (party 1, *ρ*_1_ = [∑_*k*=1_^*p*^‍exp⁡(*λ*_*k*_−*λ*_1_)]^−1^)				
	TW^c^	FP^d^	ANAP^d^	ROP^e^
Central Est.^a^ of *ρ*_1_ (conf. Int.^b^)	*ρ*_TW_^I^ = 0.014 (0.006, 0.034)	*ρ*_FP_ = 0.08 (0.046, 0.145)	*ρ*_ANAP_^T^ = 0.08 (0.038, 0.133)	*ρ*_ROP_^P^ = 0.007 (0.002, 0.022)

Convergence coefficient (*c* ≡ *c*(***λ***, *β*, *σ* ^2^) = 2*β*[1 − 2*ρ*_1_]*σ* ^2^)				
Central Est.^a^ of *c* (conf. Int.^b^)	4.06 (3.474, 4.579)	1.49 (0.675, 2.234)	5.75 (4.388, 7.438)	5.99 (5.782, 7.833)

^a^Central Est.: central estimate.

^b^Conf. Int.: confidence intervals.

^c^Israel: TW: Third Way.

^d^Turkey: DYP: True Path Party.

^e^Poland: ROP: Movement for Reconstruction of Poland.

**Table 5:** MNL spatial model in anocracies.

	Georgia^c^		Russia^b^		Azerbaijan^d^	
	Party	2008	Party	2007	Party	2010
Var		Est.^a^ |t − value|		Est.^a^ |t − value|		Est.^a^ |t − value|

*β*		0.78*** (13.78)		0.181*** (12.08)		1.34*** (4.62)

Valance	*λ*_S_	2.56*** (13.66)	*λ*_CPRF_	1.971*** (17.79)	*λ*_YAP_	1.30* (2.14)
	*λ*_G_	1.50*** (7.96)	*λ*_LDRP_	0.153 (1.09)		
	*λ*_P_	0.53* (2.51)	*λ*_SR_	−0.404*** (2.50)		

Base party		N		ER		AXCP-MP
*n*		676		1004		149
*LL*		−533		−797		−11.5

^a∗^prob < 0.05; **prob < 0.01; ***prob < 0.001.

^b^Georgia: S: Saakashvili, G: Gachechiladze, P: Patarkatsishvili, and N: Natelashvili.

^c^Rusia: ER: United Russia; CPRF: Communist Party; SR: Fair Russia; LDPR: Liberal Democratic Party.

^d^Azerbaijan: YAP: Yeni Azerbaijan Party AXCP-MP: Azerbaijan Popular Front Party (AXCP)-and Musavat (MP).

**Table 6:** The convergence coefficient in anocracies.

	Georgia	Russia	Azerbaijan^d^
	2008	2007	2010
Weight of policy differences (*β*)			
Est. *β* (conf. Int.^a^)	0.78 (0.66, 0.89)	0.181 (0.15, 0.20)	1.34 (0.77, 1.91)
	Electoral variance (trace∇ = *σ* ^2^)		
*σ* ^2^	1.73	5.90	0.93

Probability of voting for lowest valence party (party 1, *ρ*_1_ = [∑_*k*=1_^*p*^‍exp⁡(*λ*_*k*_−*λ*_1_)]^−1^)			
	N^c^	SR^b^	AXCP-MP^d^
Est. *ρ*_1_ (conf. Int.^a^)	*ρ*_N_^G^ = 0.05 (0.03, 0.07)	*ρ*_SR_^R^ = 0.07 (0.04, 0.12)	*ρ*_AXCP-MP_ = 0.21 (0.08, 0.47)

Convergence coefficient (*c* ≡ *c*(***λ***, *β*, *σ* ^2^) = 2*β*[1 − 2*ρ*_1_]*σ* ^2^)			
Est. *c* (conf. Int.^a^)	2.42 (1.99, 2.89)	1.83 (1.35, 2.28)	1.44 (0.085, 2.984)

^a^Conf. Int.: confidence intervals.

^b^Georgia: N: Natelashvili.

^c^Russia: SR: Fair Russia.

^d^Azerbaijan: AXCP-MP: Azerbaijan Popular Front Party (AXCP) and Musavat (MP).

The estimates for Azerbaijan are less precise because the sample is small.

**Table 7:** Convergence and fragmentation.

	Plurality systems					
Variable	US				Britain	
Political system		Presidential			Parliamentary	
Election year	2000	2004	2008		2005	2010
Conv. Coef.^a^ (conf. Int.^b^)	0.38 (0.2, 0.7)	0.45 (0.2, 0.8)	1.11 (0.7, 1.5)		0.84 (0.5, 1.3)	0.95 (0.9, 1.1)
Converge to mean	Yes	Yes	Yes		Yes	Yes
Number of parties^c^	2	2	2		9	9

		President				
*env* ^ c^	2.16	2.05	2.05			

		House of Representatives			House of Commons	
*env* ^ d^	2.25	2.18	2.18		3.61	3.74
*ens* ^ d^	2.02	2.00	2.00		2.47	2.58

	Proportional Representation					
	Israel		Turkey		Poland	
Political system	Fragmented		Fragmented	Cut off	Fragmented	
Election year	1996		1999	2002	1997	
Conv. Coef.^a^ (conf. Int.^b^)	3.98 (3.5, 4.6)		1.49 (0.7, 2.2)	5.94 (4.4, 7.4)	6.82 (5.8, 7.8)	
Converge to mean	No		Likely	No	No	
Number of parties^b^	11		9	10	7	

	Prime Ministers^e^					
*env* ^ c^	2.00					

	Knesset		Parliament		Sejm	
*env* ^ c^	5.84		6.91	5.62	4.99	
*ens* ^ c^	5.89		6.35	2.29	6.77	

	Anocracies—plurality					
	Georgia		Russia		Azerbaijan	
Political system	Presidential		Presidential		Presidential	
Election year	2008		2007		2010	
Conv. Coef.^a^ (conf. Int.^b^)	2.42 (2.0, 2.9)		1.83 (1.4, 2.3)		1.44 (0.1, 3.0)	
Converge to mean	No		Likely		No	

	President		President (2008)		President (2008)	
Number of parties^c^	8		4		7	
*env* ^ d^	2.76		1.88		1.31	

	Parliamentary		Duma (2007)		National assembly (2010)	
Number of parties^a^	5		7		12	
*env* ^ d^	2.56		2.22		4.74	
*ens* ^ d^	1.55		1.94		2.27	

^a^This is the central estimate of the convergence coefficient.

^b^Conf. Int.: confidence interval rounded to the nearest tenth.

^c^Number of parties who won votes in the election.

^d^Based on the number of parties who obtained seats in the election.

^e^This was the first time the Prime Minister was elected on a ballot separate from the Knesset.

## Appendix

### A. Confidence Intervals

Schofield's [9] Valence Theorem, presented in Section 2, perfectly predicts whether parties converge to or diverge from the electoral origin. Convergence or divergence depends on the value of the convergence coefficient *c* ≡ 2*β*[1 − 2*ρ*_1_]*σ* ^2^ in (15) and on the Characteristic matrix of party 1 with lowest valence, *C*_1_ = 2*β*(1 − 2*ρ*_1_)∇−*I* in (17). Both *c* and *C*_1_ depend on *β* and on *ρ*_1_ = [∑_*k*=1_^*p*^exp⁡(*λ*_*k*_−*λ*_1_)]^−1^ in (14).

The central estimate of *β* and of ***λ*** = (*λ*_1_,…, *λ*_*p*_) given by the MNL regressions depend on the sample of voters surveyed as do *ρ*_1_, *c*, and *C*_1_. Thus, to make inferences from empirical models we need the 95% confidence bounds of these estimates. Using these bounds we assert with some degree of certainty whether parties converge to or diverge from the electoral mean or if there is a knife-edge unstable equilibrium.

To build these bounds, we could perform simulations of the election. For each simulation we could generate the value of *β*, ***λ*** = (*λ*_1_,…, *λ*_*p*_), *ρ*_1_, *c* and *C*_1_. Repeating the simulation many times would generate their distribution from which we could derive their 95% confidence bounds. Note that *c* and *C*_1_ increase in *β* and decrease in *ρ*_1_. So that given the electoral covariance matrix ∇ and variance/trace *σ* ^2^ in (16) of an election, when in a simulation *β* has a low value and *ρ*_1_ a high one, the values of *c* and *C*_1_ are low with the opposite being true when *β* is high and *ρ*_1_ is low. Since we have not performed simulations for the elections in this study, we use these features of *c* and *C*_1_ to generate our confidence bounds.

Let *L* identify the lower and *U* the upper bounds of the 95% confidence intervals of any estimate. The MNL estimation for an election gives the confidence bounds of *β* and *λ*_1_, (*β* ^*L*^, *β* ^*U*^) and [*λ*_1_^*L*^, *λ*_1_^*U*^]. To estimate the bounds on *ρ*_1_ in (14), [*ρ*_1_^*L*^, *ρ*_1_^*U*^], we use the bounds on *λ*_1_ and Taylor's Theorem, which asserts that

(A.1) ρ1(λ1±h)=ρ1(λ1)±hdρ1dλ1=ρ1(λ1)±hρ1(λ1)[1−ρ1(λ1)]=ρ1(λ1)[1±h(1−ρ1(λ1))]=[ρ1L,ρ1U].

Using (15) and the bounds on *β* and *ρ*_1_ we build the confidence intervals for the convergence coefficient *c* as follows. In (15) use *β* ^*L*^ and *ρ*_1_^*U*^ to get the lower bound of *c*, *c* ^*L*^, and use *β* ^*U*^ and *ρ*_1_^*L*^ for the upper bound of *c*, *c* ^*U*^. The 95% confidence interval of the convergence coefficient is then

(A.2) $$\begin{array}{c} \left[c^{L},c^{U}\right]=\left[2\beta^{L}\left[1-2\rho_{1}^{U}\right]\sigma^{2},2\beta^{U}\left[1-2\rho_{1}^{L}\right]\sigma^{2}\right]. \end{array}$$

Following a similar procedure we estimate the bounds for *C*_1_ using (17) and the corresponding bounds of *β* and *ρ*_1_ to get the bounds for the Hessian of the lowest valence party

(A.3) $$\begin{array}{c} \left[C_{1}^{L},C_{1}^{U}\right]=\left[2\beta^{L}\left[1-2\rho_{1}^{U}\right]\nabla -I,2\beta^{U}\left[1-2\rho_{1}^{L}\right]\nabla -I\right]. \end{array}$$

Clearly, the bounds for *c* and *C*_1_ must be similar to those generated by repeated simulations.

Using these procedures, we now derive the 95% confidence intervals for the central estimates of *ρ*_1_, *c*, and *C*_1_ for each of the elections studied (see summary in Tables 2, 4, and 6). We first derive the detail of the confidence bounds for the 2000 US election, then in less detail those of other elections.  Table 7 gives the values needed to derive the confidence intervals for the convergence coefficient of the election.

#### A.1. Convergence in Plurality Systems

##### A.1.1. Confidence Bounds for the 2000, 2004, and 2008 US Elections

*US 2000 Election*. From Table 1, the 95% confidence interval for *β*_2000_^US^ = 0.82 are [*β*_2000_^US*L*^,  *β*_2000_^US*U*^] = [0.82 ± 1.96 × 0.06] = [0.71,0.93]. Using (A.1), the bounds for *ρ*_rep_^US2000^ = 0.4 in (20) are [*ρ*_rep_^US2000*L*^,  *ρ*_rep_^US2000*U*^] = [0.35, 0.44]. Using these bounds and (18), the bounds for the convergence coefficient for the 2000 US election in (21) from (A.2) are

(A.4) [c2000USL,c2000USU]  =[2(0.71)(1−2×0.44)(1.17),2(0.93)(1−2×0.35)(1.17)]  =[0.20,0.65].

With 95% confidence, the convergence coefficient is below 1 meeting the sufficient and thus necessary condition for convergence to the mean. The bounds on Bush's characteristic matrix in (22) from (A.3) are

(A.5) [CrepUS2000L,CrepUS2000U] =[2(0.71)(1−2×0.44)[0.58−0.20−0.200.59]−I, 2(0.93)(1−2×0.35)[0.58−0.20−0.200.59]−I] =[[−0.90−0.03−0.03−0.90],[−0.68−0.11−0.11−0.67]].

Since the eigenvalues of the lower and upper bounds of *C*_rep_^US2000^ are negative [*C*_rep_^US2000*L*^ = (−0.87, −0.93),  *C*_Bush_^US2000*U*^ = (−0.79, − 0.57)], with 95% confidence Bush's vote share is at a maximum when all parties locate at the mean. Thus, with a high degree of certainty the origin is a LNE for the 2000 US election.

*US 2004 Election*. From Table 1, the 95% confidence bounds of *β*_2004_^US^ = 0.95 is [*β*_2004_^US*L*^,  *β*_2004_^US*U*^] = [0.95 ± 1.96 × 0.07] = [0.82,1.08]. Using (A.1), the bounds of *ρ*_rep_^US2004^ = 0.4 in (25) are [*ρ*_rep_^US2004*L*^,  *ρ*_rep_^US2004*U*^] = [0.35,0.44]. The bounds for *c*_2004_^US^ = 0.38 in (21) from (A.2) and for the characteristic matrix of Bush *C*_rep_^2004^ in (27) from (A.3) are

(A.6) [c2004USL,c2004USU]=[2(0.82)(1−2×0.44)(1.17),2(1.08)(1−2×0.35)(1.17)]=[0.23,0.76],[CrepUS2004L,CrepUS2004U] =[2(0.82)(1−2×0.44)[0.58−0.18−0.180.59]−I,2(1.08)(1−2×0.35)[0.58−0.18−0.180.59]−I] =[[−0.89−0.04−0.04−0.88],[−0.62−0.12−0.12−0.62]].

The convergence coefficient is significantly below 1. Bush maximizes his vote share when located at the origin since the eigenvalues of the lower and upper bounds of *C*_rep_^US2004^ are negative [*C*_rep_^US2004*L*^ = (−0.87, −0.93),  *C*_rep_^US2004*U*^ = (−0.79, −0.57)]. Thus, with 95% confidence Bush does not want to move from the mean implying that with a great certainty the origin is a LNE for the 2004 US election.

*US 2008 Election*. From Table 1 the bounds of *β*_2008_^US^ = 0.85 are [*β*_2008_^US*L*^,  *β*_2008_^US*U*^] = [0.85 ± 1.96 × 0.06] = [0.73,  0.97]. Using (A.1), those of *ρ*_rep_^US2008^ in (30) are [*ρ*_rep_^US2008*L*^,  *ρ*_rep_^US2080*U*^] = [0.26,0.35]. So that the bounds for c_2008_^US^ = 1.1 in (31) from (A.2) and for McCain's characteristic matrix C_rep_^US2008^ in (32) from (A.3) are

(A.7) [c2008USL,c2008USU]=[2(0.73)(1−2×0.35)(1.63),2(0.97)(1−2×0.26)(1.63)]=[0.71,1.52],[CrepUS2008L,CrepUS2008U]=[2(0.73)(1−2×0.35)[0.80−0.13−0.130.83]−I,2(0.97)(1−2×0.26)[0.80−0.13−0.130.83]−I]=[[−0.65−0.06−0.06−0.64],[−0.26−0.12−0.12−0.23]].

The convergence coefficient is not statistically different from 1 and thus meets the necessary but not the sufficient condition for convergence. Since the eigenvalues of the lower and upper bounds of *C*_rep_^US2008^ are negative  [*C*_rep_^US2008*L*^ = (−0.75, −0.59),  *C*_rep_^US2008*U*^ = (−0.37, −0.12)], then with 95% confidence McCain stays at the origin. With a high degree of certainty the mean is an LNE for the 2008 US election.

##### A.1.2. Confidence Bounds for the 2005 and 2010 UK Elections

*UK 2005 Election*. From Table 1, the bounds of *β*_2005_^UK^ = 0.15 are [*β*_2005_^UK*L*^,  *β*_2005_^UK*U*^] = [0.15 ± 1.96 × 0.01] = [0.13,0.17]. Using (A.1), those for *ρ*_lib_^UK2005^ in (35) are [*ρ*_lib_^UK2005*L*^,  *ρ*_lib_^UK2005*U*^] = [0.18,0.32], so that those for *c*_2005_^UK^ in (36) from (A.2) and for the Liberal Democrats' characteristic matrix *C*_lib_^UK2005^ in (37) from (A.3) are

(A.8) [c2005UKL,c2005UKU]=[2(0.13)(1−2×0.32)(5.61),2(0.17)(1−2×0.18)(5.61)]=[0.51,1.25],[ClibUK2005L,ClibUK2005U]=[2(0.13)(1−2×0.32)[1.650.000.003.96]−I,2(0.17)(1−2×0.18)[1.650.000.003.96]−I]=[[−0.850.000.00−0.64],[−0.630.000.00−0.12]].

With *c*_2005_^UK^ not significantly different from 1, the necessary but not the sufficient condition for convergence to the mean has been met. The eigenvalues of the bounds on *C*_lib_^UK2005^ are negative, [*C*_lib_^UK2005*L*^ = (−0.85, −0.64), *C*_lib_^UK2005*U*^ = (−0.37, −0.12)]. With 95% confidence the LibDem locate at the origin and the mean is an LNE of the 2005 UK election.

*UK 2010 Election.* From Table 1, the bounds of *β*_2010_^UK^ = 0.86 are [*β*_2010_^UK*L*^,  *β*_2010_^UK*U*^] = [0.86 ± 1.96 × 0.02] = [0.81, 0.90]. Using (A.1), those for *ρ*_lab_^UK2010^ in (40) are [*ρ*_lab_^UK2010*L*^,  *ρ*_lab_^UK2010*U*^] = [0.29, 0.32]. So that those for *c*_UK_^2010^ in (41) from (A.2) and for Labour's characteristic matrix *C*_lab_^UK2010^ in (42) from (A.3) are

(A.9) [cUK2010L,cUK2010U]=[2(0.81)(1−2×0.32)(1.46),2(0.90)(1−2×0.29)(1.46)]=[0.86,1.10],[ClibUK2010L,ClibUK2010U]=[2(0.81)(1−2×0.32)[0.600.070.070.86]−I,2(0.90)(1−2×0.29)[0.600.070.070.86]−I]=[[−0.650.040.04−0.49],[−0.550.050.05−0.35]].

The convergence coefficient meets the necessary but not the sufficient condition for convergence to the mean as is not significantly different from 1. The eigenvalues of the bounds of *C*_lib_^UK2010^ are negative, [*C*_lab_^UK2010*L*^ = (−0.66, −0.48), *C*_lab_^UK2015*U*^ = (−0.56, −0.34)]. Thus, with 95% confidence Labour does not want to move from the origin and the origin is an LNE of the model of the 2010 UK election.

#### A.2. Convergence in Proportional Systems

##### A.2.1. Confidence Bounds for the 1996 Israeli Election

From Table 3, the bounds of *β*_1996_^I^ = 1.207 are [*β*_1996_^I*L*^, *β*_1996_^I*U*^] = [1.207 ± 1.96 × 0.065] = [1.076,1.338]. Using (A.1), those for *ρ*_TW_^I1996^ in (45) are [*ρ*_TW_^I1996*L*^,  *ρ*_TW_^I1996*U*^] = [0.006,0.034], implying that those of *c*_1996_^I^ in (46) from (A.2) and for the TW's characteristic matrix *C*_TW_^I1996^ in (47) from (A.3) are

(A.10) [c1996IL,c1996IU]=[2(1.076)(1−2×0.034)(1.732),2(1.338)(1−2×0.006)(1.732)]=[3.474,4.579],[CTWI1996L,CTWI1996U]=[2(1.076)(1−2×0.034)[1.000.5910.5910.732]−I,2(1.338)(1−2×0.006)[1.000.5910.5910.732]−I]=[[1.0061.1851.1850.468],[1.6441.5631.5630.935]].

Since *c*_1996_^I^ is significantly greater than 2, the necessary condition for convergence to the electoral mean is *not* met. The lower and upper bounds of *C*_TW_^I1996^ have one negative and one positive eigenvalue, [*C*_*TW*_^I1996*L*^ = (−0.48,1.95), *C*_TW_^I1996*U*^ = (−0.313,2.892)], TW is at a saddle point at both bounds. Thus, with 95% confidence TW locates away from the origin and the origin *fails* to be a LNE for the 1996 Israeli election.

##### A.2.2. Confidence Bounds for the 1999 and 2002 Turkish Elections

*1999 Turkish Election*. From Table 3, the bounds of *β*_1999_^T^ = 0.375 are [*β*_1999_^T*L*^,  *β*_1999_^T*U*^] = [0.375 ± 1.96 × 0.088] = [0.203,0.547]. Using (A.1), those for *ρ*_FP_^T1999^ in (50) are [*ρ*_FP_^T1999*L*^,  *ρ*_FP_^T1999*U*^] = [0.046,0.145], so that those of *c*_1999_^T^ in (51) from (A.2) and for the FP's characteristic matrix *C*_FP_^T1999^ in (52) from (A.3) are

(A.11) [c1999TL,c1999TU]=[2(0.203)(1−2×0.145)(2.34),2(0.547)(1−2×0.046)(2.34)]=[0.675,2.234],[CFPT1999L,CFPT1999U]=[2(0.203)(1−2×0.145)[1.200.780.781.14]−I,2(0.547)(1−2×0.046)[1.200.780.781.14]−I]=[[−0.6540.2250.225−0.671],[0.1920.7750.7750.132]].

Since *c*_1999_^T^ is significantly greater than 2, the necessary condition for convergence to the mean is *not* met. *C*_FP_^T1999*L*^ has two negative eigenvalues, [*C*_FP_^T1999*L*^ = (−0.888, −0.437)] indicating that at the lower bound FP has no incentive to move from the origin. However, *C*_FP_^T1999*U*^ has one negative and one positive eigenvalue *C*_FP_^T1999*U*^ = (−0.614,0.938); thus, FP is at a saddlepoint at the upper bound and wants to move from the mean. At the central estimate of *C*_FP_^T1999^ given in (52) FP is also at a saddlepoint. It is more probable that FP wants to move and that the electoral mean is *not* a LNE of 1999 Turkish election.

*2002 Turkish Election*. From Table 3, the bounds of *β*_2002_^T^ = 1.52 are [*β*_2002_^T*L*^, *β*_2002_^T*U*^] = [1.52 ± 1.96 × 0.12] = [1.285,1.755]. Using (A.1), those for *ρ*_ANAP_^T2002^ in (55) are [*ρ*_ANAP_^T2002*L*^,  *ρ*_ANAP_^T2002*U*^] = [0.038,0.133], implying that those of *c*_2002_^T^ in (56) from (A.2) and for the ANAP's characteristic matrix *C*_ANAP_^T2002^ in (57) from (A.3) are

(A.12) [c2002TL,c2002TU]=[2(1.285)(1−2×0.133)(2.33),2(1.755)(1−2×0.038)(2.33)]=[4.338,7.438],[CANAPT2002L,CANAPT2002U]=[2(1.285)(1−2×0.133)[1.180.740.741.15]−I,2(1.755)(1−2×0.038)[1.180.740.741.15]−I]=[[−0.6600.2130.213−0.669],[0.1720.7350.7350.142]].

Since *c*_2002_^T^ is significantly greater than 2, the necessary condition for convergence to the mean has *not* been met. The eigenvalues of *C*_ANAP_^T2002*L*^ are all negative, *C*_ANAP_^T2002*L*^ = (−0.878, −0.451) so that at the lower bound ANAP remain at the mean. However, at *C*_ANAP_^T2002*U*^ there is one negative and one positive eigenvalue *C*_ANAP_^T2002*U*^ = (−0.578,0.892), ANAP is at a saddlepoint and wants to move. At the central estimate of *C*_ANAP_^T2002^ in (57) the eigenvalues are both positive and ANAP is minimizing its vote share. There is a high likelihood that ANAP wants to move from the origin and that the electoral mean is *not* a LNE of 2002 Turkish election.

##### A.2.3. Confidence Bounds for the 1997 Polish Election

From Table 3, the bounds of *β*_1997_^P^ = 1.739 are [*β*_1997_^P*L*^,  *β*_1997_^P*U*^] = [1.739 ± 1.96 × 0.12] = [1.512, 1.966]. Using (A.1), those for *ρ*_UPR_^P1997^ in (60) are [*ρ*_1997_^P*L*^,  *ρ*_1997_^P*U*^] = [0.002, 0.022], so that those of *c*_1997_^P^ in (61) from (A.2) and for the UPR's characteristic matrix *C*_UPR_^P1997^ in (62) from (A.3) are

(A.13) [c1997PL,c1997PU]=[2(1.512)(1−2×0.022)(2),2(1.966)(1−2×0.002)(2)]=[5.782,7.833],[C1997PL,C1997PU]=[2(1.512)(1−2×0.022)[1001]−I,2(1.966)(1−2×0.002)[1001]−I]=[[1.8910.0000.0001.891],[2.9160.0000.0002.916]].

With *c*_1997_^P^ significantly greater than 2, the necessary condition for convergence to the mean is *not* met. The eigenvalues of the bounds of *C*_1997_^P^ are positive, [*C*_UPR_^P1997*L*^ = (1.891,  1.891), *C*_UPR_^P1997*L*^ = (2.916,  2.916)] as are those of the central estimate of *C*_1997_^P^ in (62). Thus, with a high probability UPR will not locate at the mean and the electoral mean is *not* a LNE of 1997 Polish election.

#### A.3. Convergence in Anocracies

##### A.3.1. Confidence Bounds for the 2008 Georgian Election

From Table 5, the bounds of *β*_2008_^G^ = 0.78 are [*β*_2008_^G*L*^,  *β*_2008_^G*U*^] = [0.78 ± 1.96 × 0.06] = [0.66, 0.89]. Using (A.1), those for *ρ*_N_^G2008^ = 0.05 in (65) are [*ρ*_N_^G200*L*8^,  *ρ*_N_^G2008*U*^] = [0.03, 0.07]. So that those of *c*_2008_^G^ in (66) from (A.2) and for Natelashvili's characteristic matrix *C*_N_^G2008^ in (67) from (A.3) are

(A.14) [c2008GL,c2008GU]=[2(0.66)(1−2×0.07)(1.73),2(0.89)(1−2×0.03)(1.73)]=[1.99,2.89],[CNG2008L,CNG2008U]=[2(0.66)(1−2×0.07)[0.820.030.030.91]−I,2(0.89)(1−2×0.03)[0.820.030.030.91]−I]=[[−0.060.030.030.05],[0.370.050.050.52]].

Since *c*_2008_^G^ is *not* statistically different from 2, the necessary condition for convergence is *not* met. The lower bound of *C*_N_^G2008^ has one negative and one positive eigenvalue [*C*_N_^G2008*L*^ = (−0.068,0.058)], so that at the lower bound Natelashvili's vote share function is at a saddlepoint. The upper bound has two positive eigenvalues [*C*_N_^G200*U*^ = (0.355,0.535)] so that at the upper bound Natelashvili is minimizing his vote share. At the central estimate of *C*_N_^G2008^ in (67) Natelashvili is also minimizing his vote share. Thus, with a high probability Natelashvili diverges from the mean and the mean *cannot* be a LNE of the 2008 Georgian election.

##### A.3.2. Confidence Bounds for the 2007 Russian Election

From Table 5, the bounds of *β*_2007_^R^ = 0.181 are [*β*_2007_^R*L*^,  *β*_2007_^R*U*^] = [0.18 ± 1.96 × 0.01] = [0.15,0.20]. Using (A.1), those for *ρ*_SR_^R2007^ = 0.07 in (70) are [*ρ*_SR_^R2007L^,  *ρ*_SR_^R2007*U*^] = [0.04, 0.12]. So that those of *c*_2007_^R^ in (71) from (A.2) and for SR's characteristic matrix *C*_SR_^R2007^ in (72) from (A.3) are

(A.15) [c2007RL,c2007RU]=[2(0.15)(1−2×0.12)(5.9),2(0.15)(1−2×0.04)(5.9)]=[1.35,2.28],[CSRR2007L,CSRR2007U]=[2(0.15)(1−2×0.12)[2.950.130.132.95]−I,2(0.2)(1−2×0.04)[2.950.130.132.95]−I]=[[−0.330.030.03−0.33],[0.140.050.050.14]].

With *c*_2007_^R^ not significantly different from 2, the necessary for convergence is *not* met. The lower bound of *C*_SR_^R2007^ has two negative eigenvalues, [*C*_SR_^R2007*L*^ = (−0.30, − 0.36)] implying that at lower bound SR's vote share is at a maximum and SR stays at the origin. However, at the upper bound there are two positive eigenvalues, [*C*_SR_^R2007*U*^ = (0.09, 0.19)]. Thus at the upper bound SR's vote share is at minimum and SR wants to move. At the central estimate of *C*_SR_^R2007^ in (72) SR also has two negative eigenvalues suggesting that SR wants to remain at the origin. So it seems more likely that SR will stay at the origin and that the mean is a LNE of the 2007 Russian election.

##### A.3.3. Confidence Bounds for the 2010 Azerbaijani Election

From Table 5 the bounds for *β*_2010_^A^ = 1.34 are [*β*_2010_^A*L*^,  *β*_2010_^A*U*^] = [1.34 ± 1.96 × 0.29] = [0.77, 1.91]. Using (A.1), those for *ρ*_AXCP-MP_^A2010^ = 0.21 in (75) are [*ρ*_AXCP-MP_^A2010*L*^,  *ρ*_AXCP-MP_^A2010*U*^] = [0.08, 0.47]. So that those of *c*_2010_^A^ in (76) from (A.2) and for AXCP-MP's characteristic matrix *C*_AXCP-MP_^A2010^ in (77) from (A.3) are

(A.16) [c2010AL,c2010AU]=[2(0.77)(1−2×0.47)(0.93),2(1.91)(1−2×0.08)(0.93)]=[0.085,2.984],[CAXCP-MPA2010L,CAXCP-MPA2010U]=[2(0.77)(1−2×0.47)(0.445)−1,2(1.91)(1−2×0.08)(0.445)−1]=[0.037,1.428].

With *c*_2010_^A^ not significantly different from 1, the dimension of the policy space, the necessary and the sufficient (in this case the same) conditions for convergence are *not* met. This one-dimensional characteristic matrix has positive eigenvalues at the lower and upper bounds as does the central estimate of *C*_AXCP-MP_^A2010^ = 0.445 in (77). It is then very likely that AXCP-MP locates far from the origin and that the electoral mean is *not* an LNE for the 2010 election in Azerbaijan.
